# Pediatric assessment and early resuscitation capacity building: Multi-country implementation of the StART Training-of-Trainers Model in Rwanda, Uganda, and Nigeria

**DOI:** 10.1371/journal.pgph.0006137

**Published:** 2026-08-14

**Authors:** Rona Breese, Jean de Dieu H. Tuyishime, Ibrahim Salisu, Bob Okeyo, Adamson Phiri, Amos Zacharia, Amelia Breese, Thomas Cooney, Emily Whalen, Anne Mulwa, Lilian Macharia, Elisha Mullen Okaisu, Rebecca Silvers

**Affiliations:** 1 Independent Training Consultant, Dar es Salaam, Tanzania; 2 University of Rwanda, College of Medicine and Pharmacy, School of Health Sciences, Kigali, Rwanda; 3 Department of Anesthesia and Intensive Care Federal Teaching Hospital, Katsina- Nigeria; 4 AIC Kijabe Hospital, Kijabe, Kenya; 5 Université Iba der Thiam de Thiès, Thiès, Senegal; 6 Mbeya Zonal Referral Hospital/ University of Dar es salaam- Mbeya College of Health and Allied Sciences, Mbeya, Tanzania; 7 Royal Hobart Hospital, Hobart, Tasmania, Australia; 8 Center for Global Nursing at University of California San Francisco, San Francisco, California, United States of America; 9 St. Paul’s University, Limeru, Kenya; 10 CURE Children’s Hospital of Kenya, Kijabe, Kenya; 11 CURE Children’s Hospital of Uganda, Mbale, Uganda; PLOS: Public Library of Science, UNITED STATES OF AMERICA

## Abstract

Child mortality remains marked by profound inequities in sub-Saharan Africa, where many deaths occur within the first 24 hours of hospital admission due to delays in recognizing and managing critical illness. Access to practical, context-appropriate pediatric resuscitation training is limited, contributing to disparities in the quality of emergency care. The Systematic Assessment Resuscitation Training (StART) course was developed to address this gap through a one-day, multidisciplinary pediatric Basic Life Support (BLS) program with an embedded Training-of-Trainers (ToT) model, promoting sustainability. A mixed-methods pilot evaluation was conducted in three African countries: Uganda, Nigeria, and Rwanda. Quantitative data from pre- and post-course multiple-choice tests and BLS skills assessments measured knowledge and competence. Immediate and follow-up surveys captured participants’ perceptions of relevance and confidence, as well as self-reported application of learning in practice. Qualitative data from open-ended responses were analyzed thematically. A total of 134 participants completed the StART course, including nurses, doctors, and anesthesia providers, with 14 completing the ToT program. Knowledge scores increased significantly across all sites (mean improvement 3.4 points on a 15-point test scale; p < 0.0001), with all participants meeting BLS competency standards. Gains were consistent across professions and countries. Follow-up surveys (response rate 67%) indicated participant-reported application of learning in clinical care, with perceived improvements in confidence and teamwork. Trainers described the ToT model as practical and achievable within local resource constraints. The StART pediatric resuscitation course was feasible, acceptable, and effective across diverse African hospital contexts. It demonstrated measurable learning gains and practical competence, alongside early participant-reported indications of improvements in teamwork and application of learning in clinical practice. The ToT model presents a promising approach to sustainable, locally led resuscitation training. Further evaluation is needed to assess long-term skill retention, institutional uptake, and impact on patient outcomes.

## Introduction

### Background

Pediatric mortality remains a pressing global health issue, and rates across the globe continue to fall short of the United Nations Sustainable Development Goal (SDG), which aims to reduce deaths among children under five years old. An estimated 4.9 million children under the age of five die each year, with the majority of these deaths occurring in low- and middle-income countries (LMICs), particularly in sub-Saharan Africa (SSA) [1]. Mortality is strongly influenced by the availability, accessibility, and quality of care delivered across a country’s health system [2]. In many hospitals in SSA, shortages of skilled pediatric providers and limited availability of essential resources contribute to preventable child deaths [3, 4].

Additionally, persistent inequities in triage systems and gaps in provider training hinder the timely recognition and management of critical illness [5]. These challenges are particularly acute in sub-Saharan Africa, where under-five mortality remains the highest globally, even though many life-threatening conditions are preventable or treatable with timely and appropriate care [1]. In many of these settings, limited patient monitoring capacity, staff shortages, and challenges implementing early warning systems hinder timely recognition of deterioration allowing otherwise manageable conditions to progress to critical illness or death [6]. Hospital-based data from low and middle-income countries show that a substantial proportion of pediatric in-hospital deaths occur within the first twenty-four to forty-eight hours of admission [7]. This pattern underscores the pressing need to enhance the early recognition and management of critical illnesses. Although early mortality in these settings is influenced by multiple factors, including travel time and long waits for care, the ability of frontline providers to recognize early signs of deterioration and begin timely management can significantly reduce the risk of pediatric emergencies and preventable deaths [8].

There is growing international recognition that the identification and treatment of critical illness must serve as a foundational component of acute care systems in low- and middle-income countries (LMICs), as emphasized by the World Health Organization (WHO) and the Essential Emergency and Critical Care (EECC) framework [9,10]. The World Health Assembly Resolution 76.2 further reinforces this global priority by urging Member States to strengthen and integrate emergency, critical, and operative care systems as essential elements of universal health coverage [11]. The EECC framework highlights the importance of early recognition and management of critical illness as an equity-driven priority for reducing preventable deaths. However, once deterioration occurs, effective resuscitation becomes essential for survival [12]. Training in Basic Life Support (BLS) is therefore a critical element in strengthening the continuum of emergency and critical care capacity and improving outcomes across diverse health systems.

Several well-established courses offer pediatric basic life support (BLS) training and are delivered through national and international organizations, including the American Heart Association (AHA), the European Resuscitation Council (ERC), and the UK Resuscitation Council (UKRC). These programs are based on international consensus guidelines developed by the International Liaison Committee on Resuscitation [12], which review scientific evidence to guide best practice in resuscitation and emergency cardiovascular care. BLS courses are designed to equip participants with the skills to recognize and respond appropriately to cardiac arrest and other life-threatening emergencies. In hospital settings, such training improves response times, the quality of resuscitation efforts, and survival outcomes, while also enhancing providers’ confidence and readiness in emergency situations [13].

Despite their global reach, conventional Basic Life Support (BLS) programs are designed primarily for high-resource environments and are often not relevant or transferable to the realities of low- and middle-income country (LMIC) health systems. In these contexts, differences in infrastructure, workforce composition, and available equipment limit the applicability of standardized algorithms and simulation-based training models developed for high-income settings. Although numerous emergency and resuscitation training programs have been introduced across LMICs, their effectiveness in changing clinical practice or improving patient outcomes within these environments has not been systematically evaluated [14]. As a result, healthcare providers may find it difficult to translate such training into daily clinical practice, thereby reducing its long-term impact on patient outcomes. These challenges are particularly evident at district and referral hospital levels in sub-Saharan Africa, where health workers face systemic constraints that impede the consistent implementation of BLS principles [5].

In response to these limitations, the World Health Organization developed the Emergency Triage Assessment and Treatment (ETAT) course to strengthen the recognition and management of critically ill children in low-resource settings. ETAT+ further expanded this model to include ongoing inpatient care beyond the initial triage phase [15]. These programs have led to important improvements in pediatric emergency response and early intervention; however, they often fall short in supporting sustained skills practice, team-based resuscitation, and institutional capacity building [16]. Without adequate tools and follow-up, many healthcare workers struggle to maintain competence and confidence in managing pediatric emergencies.

To address these gaps, the Systematic Assessment Resuscitation Training (StART) Pediatric BLS course was developed by a team of providers in sub-Saharan Africa. StART is a context-specific training program designed to build pediatric emergency response capacity, promote interdisciplinary teamwork, and support long-term skills retention. By addressing gaps left by traditional training programs, StART equips providers with essential resuscitation and assessment skills that can be used in any environment, ultimately contributing to improved care delivery and reductions in child mortality through sustainable, scalable training in resource-limited settings.

### StART pediatric BLS

StART Pediatric BLS is a one-day training that integrates pediatric assessment, early intervention, and high-quality resuscitation skills. It was co-designed by a multidisciplinary team of eight healthcare professionals from across sub-Saharan Africa (SSA), including nurses and anesthesia providers from Tanzania, Nigeria, Uganda, Kenya, and Senegal, as well as an experienced curriculum developer based in Tanzania. All contributors had extensive experience in health professions education and clinical care in resource-variable settings. Utilizing their insights into the challenges faced in SSA hospitals, such as limited staffing, equipment shortages, and high patient loads, the team developed a context-specific and feasible training program to implement within local health systems. Course design was informed by direct experience of gaps in pediatric emergency care, including delayed recognition of critical illness and limited access to resuscitation training. To ensure contextual and cultural relevance, course materials incorporate video footage (recorded with consent) from SSA hospital settings, enabling participants to recognize and respond to critically ill children similar to those they encounter in their own facilities.

Using the Airway, Breathing, Circulation, Disability, Exposure (ABCDE) approach, participants learn to systematically identify and prioritize life-threatening problems, and initiate timely interventions. Interventions include airway positioning, oxygen delivery, suctioning, bag–mask ventilation, and fluid resuscitation, as well as effective basic life support for infants and children. While resuscitation skills remain a core component, StART places equal emphasis on systematic assessment and early intervention, recognizing that many pediatric deaths arise from delays in recognizing or responding to early signs of clinical decline. By strengthening clinicians’ ability to detect and manage these conditions using available resources, StART reinforces the principle that timely intervention before arrest saves lives.

Training is designed for multidisciplinary teams of nurses, anesthesia providers, and doctors, reflecting the collaborative nature of pediatric emergency care. The course equips healthcare professionals with skills in systematic assessment, immediate management, and coordinated team response in pediatric emergencies. Its concise, one-day format minimizes the need for staff release time, making participation feasible in settings where workforce shortages limit access to multi-day training. Designed for local facility rollout, StART aims to strengthen healthcare teams and improve the quality and timeliness of pediatric emergency care across diverse SSA contexts.

A complementary two-day ToT program was developed to prepare selected participants to become local facilitators. The ToT provides additional instruction in adult learning principles and facilitation skills, scenario delivery, along with coaching during practical BLS teaching and skills testing. Participants also have the opportunity to practice teaching sessions and receive structured feedback from faculty and peers, reinforcing their confidence and instructional competence. By building local training capacity, the ToT enables hospitals to integrate StART into existing education programs and deliver regular refresher sessions that promote long-term sustainability, recognizing that resuscitation knowledge and skills decline within months without reinforcement [17,18].

### Study aims and objectives

Since the development of the StART Pediatric Resuscitation program, no formal evaluation has been conducted to assess its implementation or outcomes. This study, therefore, aimed to examine the feasibility, effectiveness, and potential sustainability of StART within African hospital contexts, focusing on how the course was received, its impact on participants’ knowledge and practice, and the potential of the ToT model to support sustainable local delivery.

The objectives were to:

- Evaluate the feasibility and acceptability of the StART pediatric resuscitation training program from the perspectives of participants and facilitators.
- Examine participant outcomes, including changes in knowledge, practical skills, confidence, and perceived application in clinical practice.
- Assess the ToT model’s role in supporting sustainable program delivery, local ownership, and capacity building within participating hospitals

## Methods

### Study design

A mixed-methods design was used to evaluate the StART Pediatric Basic Life Support (BLS) course during a multi-country pilot in Uganda, Nigeria, and Rwanda. Quantitative data measured changes in knowledge and skills while qualitative feedback captured participants’ experiences of the course, its relevance to their clinical work, and the feasibility of local implementation. Combining quantitative and qualitative data enables a deeper understanding that extends beyond knowledge acquisition, providing insight into how the course was received and applied in clinical practice.

### Setting

Three hospitals were selected for the pilot using convenience sampling: one each in Kampala (Uganda), Abuja (Nigeria), and Kigali (Rwanda). The course was offered in English; therefore, sites were chosen where English is commonly used for clinical training and communication, while acknowledging that local languages remain central in everyday clinical practice.

The hospitals represent a mix of government, private, and not-for-profit providers, varying in size, service scope, and patient volume (Table 1).

**Table 1:** Study locations, hospital sites, and key features of each participating location.

Location	Hospital Site and Key Features
Kampala, Uganda	CoRSU Hospital, Kampala: 201-bed non-profit facility, focused on elective pediatric surgical care and rehabilitation, especially for children. No dedicated Intensive Care Unit (ICU).
Abuja, Nigeria	University of Abuja Teaching Hospital (UATH): 520-bed government tertiary hospital, often functioning over capacity (>800 inpatients). Pediatric patients admitted to adult ICU.
Kigali, Rwanda	Ejo Heza Surgical Centre, Kigali: 25-bed private specialist surgical facility providing acute and elective care. No ICU.

Each country differs in pediatric health data, as reflected in under-five mortality rates and leading causes of childhood deaths. Uganda has an under-five mortality rate of 40.5 per 1,000 live births [19], with neonatal disorders, pneumonia, diarrhea, and malaria as the most common causes of death [20]. In Nigeria, the under-five mortality rate is 105 per 1,000 live births [19], and neonatal disorders, pneumonia, diarrhea, malaria, and congenital anomalies are the main contributors [20]. Rwanda’s under-five mortality rate is similar to Uganda’s at 40 per 1,000 live births [19], with neonatal disorders, pneumonia, and diarrhea as the predominant causes [20].

### Intervention

The StART Pediatric BLS course was implemented as a pilot program across three hospital sites in Uganda, Nigeria, and Rwanda between May and October 2024. Implementation consisted of three phases:

- Pre-Launch: Pilot site facilities identified training needs and formally committed to implementing the program.
- Phase 1 (Initial Training and ToT**):** External faculty delivered the first one-day StART Pediatric BLS course for up to 12 participants. This was immediately followed by a ToT for 3–6 candidates. The ToT comprised two days of teaching skills development, culminating in a full course delivered by the new trainers, with faculty present for support and mentorship.
- Phase 2 (Independent Delivery): Newly trained local trainers conducted additional StART BLS courses within their facility, extending training to other healthcare providers across pediatric, nursing, and anesthesia teams.

From the three pilots, a total of 14 participants (three to six per site) completed the ToT, preparing them to act as local trainers for future StART trainings. As part of the pilot, new trainers subsequently delivered follow-on courses at their respective hospitals, except in Rwanda, where the small facility size limited opportunities for immediate additional training.

### Participants and trainer selection

Hospital administrators nominated clinical staff who regularly care for pediatric patients. This included staff working in emergency, outpatient, pediatric medical and surgical wards, recovery (post-anesthesia care), and anesthesia services. Each course enrolled up to 12 participants to maintain safe staffing, with an emphasis on including a balanced mix of nurses, anesthesia providers, and doctors to reflect the multidisciplinary nature of pediatric care. Anesthesia providers included both physician and non-physician clinicians involved in the delivery of anesthesia care, including nurse anesthetists, as represented across the participating sites. Participants were grouped according to their primary clinical role within the hospital. A small number of staff from outside the intended professions, including three nurse assistants and one dentist, also participated in the training. Their data was retained in the overall course evaluation but excluded from profession-specific analyses. For the ToT program, candidates were selected by their hospital leadership based on their broad clinical experience, professional role and perceived ability to teach and support multidisciplinary learning. The initial trainer cohort included doctors and anesthesiologists. While nurses were not included as trainers in this pilot, they remained central to the learner group. Prior formal teaching qualifications were not required; however, selected candidates were typically experienced clinicians with an interest in education within their teams. The ToT program was designed to build facilitation and teaching capacity from this foundation, enabling trainers to deliver the course independently within their institutions.

The StART course was intentionally designed to be deliverable within low-resource hospital settings using equipment that is commonly available in such contexts. Core equipment included basic airway and resuscitation supplies such as bag–mask devices, oxygen delivery equipment, suction devices, and pediatric manikins. Manikins were selected to enable feedback on chest compressions and included infant and either child or adult models to represent a larger child. Where possible, sites used existing hospital equipment; where gaps were identified, minimal additional equipment was sourced locally to support course delivery. This approach was intended to ensure that training remained practical, context-relevant, and reproducible within participating facilities.

Training was conducted within hospital-based venues to minimize costs and maintain alignment with the clinical environment in which skills would be applied. Participants were scheduled to attend through rostered release from clinical duties, and efforts were made to ensure that they were not called back into service during training. Delivering the course on-site enabled teams to train together within their own institutional context while reducing logistical and financial barriers associated with off-site training.

### Data collection and instruments

**Fig 1:**
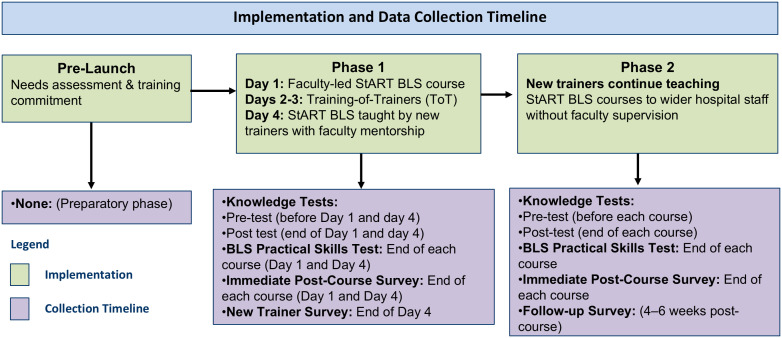
Implementation and data collection timeline for pilot StART pediatric resuscitation training.

Data were collected at all three sites at two fixed points during program implementation using various qualitative and quantitative tools (Fig 1).

Both quantitative and qualitative tools were utilized (Table 2). Quantitative data included Likert scale confidence surveys and multiple-choice knowledge tests, while qualitative data were collected through open-text feedback to explore participants’ experiences, perceptions of relevance, and views on local implementation.

**Table 2:** Quantitative and qualitative tools utilized describing the type, purpose, participants, collection method, and format of information.

Data Type	Purpose	Timing	Participants	Collection Method	Format/ Notes
**Knowledge and Skills Tests**	Pre- and post-course knowledge assessment	Pre- and post-course	All participants	MCQ paper, in-class	Quantitative (scores)
**Post-course BLS Skills Test**	Assess BLS skill competence	Post-course	All participants	Practical skills observation and assessment	Quantitative (skills checklist)
**Immediate Survey**	Course satisfaction, relevance, and immediate perceptions	End of course	All participants	Google Forms (WhatsApp link)	Mixed (Likert scale + open-text)
**Follow-up Survey**	Application in practice, barriers, ongoing value	4–6 weeks post-course	Reachable participants	Google Forms (WhatsApp link)	Mixed (Likert scale + open-text)
**Trainer Survey**	Teaching confidence, sustainability	After ToT & course delivery	New trainers	Google Forms (WhatsApp link)	Mixed (Likert scale + open-text)

### Knowledge and skills tests

Knowledge was assessed using paper-based multiple-choice (MCQ) tests administered before and after the course. The pre- and post-tests were developed as parallel forms covering the same domains: pediatric assessment, recognition of critical illness, and basic life support. To support comparability, one pilot group completed both versions prior to training and achieved similar mean scores, indicating reasonable equivalence.

In addition, a practical BLS skills assessment was conducted at the end of each course using a structured checklist. Participants demonstrated core resuscitation skills on manikins under direct observation by trainers, providing an objective measure of hands-on competence. This included recognition of cardiac arrest, chest compressions, airway management, and effective ventilation. Competency thresholds were predefined and required successful completion of all key steps in the assessment checklist, with emphasis on the quality and sequence of resuscitation actions. To support standardization across sites, course trainers conducting assessments used the same checklist and scoring criteria for competency. During the ToT phase, new trainers received demonstration, practice, feedback, and coaching in assessment delivery. This included discussion of checklist items and common scoring considerations. During initial course delivery, faculty provided oversight and mentorship, including observation of assessment processes and discussion of scoring decisions where needed, to promote consistency. Assessments were conducted by course trainers and were not blinded. Where participants did not initially meet all required criteria, they were provided with additional practice and feedback and reassessed until competency was achieved.

### Immediate post-course survey

At the end of each course, participants completed an online survey consisting of Likert-scale items and open-ended questions designed to assess satisfaction, perceived relevance, and immediate perceptions of the training.

### Four- to six-week follow-up survey

A follow-up survey was distributed electronically 4–6 weeks after training. This was used to assess participants’ satisfaction, perceived relevance of the training, and self-reported application of learning in practice.

### Trainer survey

Newly trained trainers completed a survey following course delivery. This included Likert-scale and open-ended items on teaching confidence, course quality, and anticipated benefits, to assess the acceptability and feasibility of the ToT model.

### Quantitative data analysis

Quantitative analyses were conducted using GraphPad Prism. All 134 participants completed both pre- and post-course multiple-choice question (MCQ) tests, each consisting of 15 items, allowing for paired analysis of test scores.

Descriptive statistics (mean ± standard deviation) were calculated for pre- and post-test scores. Paired t-tests were used to evaluate changes in scores where the sample size was considered sufficient (n > 30) to meet the assumption of normality based on the central limit theorem. Where subgroup sizes were smaller or distributions appeared non-normal, the Wilcoxon signed-rank test was used instead. Results were reported with corresponding p-values, and 95% confidence intervals (CIs) for mean differences where applicable. Statistical significance was set at p < 0.05.

Subgroup analyses were performed to examine score changes by country, professional role, and trainer type (faculty-led, supervised local trainers, or independent local trainers), using the same statistical approach as described above.

The effect size (paired Cohen’s d) was calculated to quantify the magnitude of the change in scores from pre- to post-training.

### Survey data analysis

Quantitative survey responses were analyzed descriptively. Likert-scale responses were summarized using counts and percentages. Where relevant, responses were stratified by country (Uganda, Nigeria, and Rwanda). Data were summarized in tables and visualized using grouped bar charts.

### Qualitative data analysis

A reflexive thematic analysis was conducted following Braun and Clarke’s six-step framework (2006). Responses to open-ended questions from the immediate post-course, follow-up, and ToT surveys were read and re-read by the authors to ensure familiarity with the data. Initial codes were generated systematically to capture meaningful features across responses.

Codes were then grouped into broader potential themes, which were reviewed, refined, and clearly defined to ensure they represented the dataset as a whole. Final themes were described and illustrated with representative participant quotations to convey the range and depth of perspectives.

### Ethical considerations

This work was conducted as part of routine pilot implementation and evaluation activities. Formal ethical approval was not sought, as the study was conducted as an evaluation of an educational training program. Participation in course assessments and evaluations was voluntary. Participants were informed verbally that participation in the evaluation was voluntary, responses would be anonymized, and non-participation would not affect course participation. Consent to participate was indicated through voluntary completion of the anonymous assessment and evaluation forms. Data were collected anonymously using Google Forms, with no personally identifiable information recorded. All data were stored securely and were accessible only to the principal investigator. Additional information regarding ethical, cultural, and scientific considerations specific to inclusivity in global research is included in the Supporting Information (S1 Checklist).

## Results

Results are presented for participant characteristics, quantitative learning outcomes, follow-up application of learning, and qualitative thematic findings.

### Participant flow and demographics

A total of 134 participants across three countries (Uganda, n = 68; Rwanda, n = 18; Nigeria, n = 48) completed the StART pediatric BLS course. All nominated participants attended.

Of those trained, 37 participants attended sessions delivered directly by faculty trainers (Uganda, n = 11; Rwanda, n = 12; Nigeria, n = 14). Fourteen of these subsequently completed the ToT program (Uganda, n = 5; Rwanda, n = 3; Nigeria, n = 6). An additional 27 participants were trained by these new trainers under direct faculty supervision (Uganda, n = 11; Rwanda, n = 6; Nigeria, n = 10). The remaining 70 participants were trained independently by the ToT trained trainers without direct faculty oversight (Uganda, n = 46; Rwanda, n = 0; Nigeria, n = 24).

All participants (100%) completed both pre- and post-course knowledge tests, providing paired data for analysis of learning outcomes. In addition, all participants completed the immediate post-course evaluation survey and met the required competency standards in the practical BLS skills assessment by the end of the course. At 4–6 weeks post-training, follow-up evaluation surveys were completed by 90 participants (Uganda, *n* = 40; Nigeria, *n* = 36; Rwanda, *n* = 14), representing an overall response rate of 67%, ranging from 56% in Uganda to 78% in Rwanda. All 14 ToT trainers completed their respective course evaluations. No participants were excluded from analysis.

A total of 134 healthcare providers participated across the three pilot sites. Most were nurses (51.5%), followed by anesthesia providers (23.9%) and doctors (17.2%). Over one-third (37.4%) had more than 10 years of professional experience. Despite this, previous exposure to pediatric emergency or resuscitation training was limited, with fewer than half reporting prior instruction in ABCDE assessment, pediatric interventions, or basic life support (Table 3).

**Table 3:** Participant demographics and prior training experience (n = 134).

Location	Uganda (n = 68)	Nigeria (n = 48)	Rwanda (n = 18)	Total (n = 134)
**Profession**				
Anesthesia provider	12 (17.7%)	14 (29.2%)	6 (33.3%)	32 (23.9%)
Anesthesia student	6 (8.9%)	0 (0%)	0 (0%)	6 (4.5%)
Doctor	7 (10.3%)	14 (29.2%)	2 (11.1%)	23 (17.2%)
Nurse	40 (58.8%)	19 (39.6%)	10 (55.6%)	69 (51.5%)
Nurse assistant	3 (4.4%)	0 (0%)	0 (0%)	3 (2.2%)
Dentist	0 (0%)	1 (2.1%)	0 (0%)	1 (0.8%)
**Years of work experience**				
< 1 year	4 (6.2%)	1 (2.1%)	0 (0%)	5 (3.8%)
1–2 years	8 (12.3%)	3 (6.3%)	1 (5.6%)	12 (9.2%)
3–5 years	15 (23.1%)	11 (22.9%)	3 (16.7%)	29 (22.1%)
6–10 years	21 (32.3%)	10 (20.8%)	5 (27.8%)	36 (27.5%)
> 10 years	17 (26.2%)	23 (47.9%)	9 (50.0%)	49 (37.4%)
**Previous pediatric training**				
ABCDE assessment	27 (41.5%)	14 (29.2%)	9 (50.0%)	50 (37.3%)
Airway/oxygen/fluid intervention	27 (41.5%)	18 (37.5%)	8 (44.4%)	53 (39.6%)
Pediatric BLS	26 (40.0%)	19 (39.6%)	8 (44.4%)	53 (40.5%)

### Knowledge and skills gains

Participants demonstrated measurable gains in knowledge across all three pilot sites, as reflected in improvements between pre- and post-course multiple-choice question (MCQ) test results. All participants successfully passed the Basic Life Support (BLS) practical skills assessment, which was conducted using a structured checklist and hands-on demonstrations on manikins observed by course trainers.

Paired pre- and post-course multiple-choice question (MCQ) test scores were available for all 134 participants (100%). The mean pre-test score was 11.1 ± 1.9 out of 15, increasing to 14.6 ± 0.6 post-test. This represents a mean improvement of 3.4 points (95% CI = 3.011–3.789), which was statistically significant (*t*(133) = 17.4, *p* < 0.0001), reflecting an overall 22.6% mean improvement in knowledge scores. The standardized effect size for this change was large (Cohen’s d = 1.5). Significant improvements were observed across all three pilot countries: in Uganda, mean scores increased from 11.3 to 14.7 (*p* < 0.0001); in Nigeria, from 11.4 to 14.5 (*p* < 0.0001); and in Rwanda, from 11.1 to 14.6 (*p* = 0.0002).

When results were analyzed by profession, anesthesia providers, medical doctors, and nurses all demonstrated statistically significant improvements in post-test scores compared with pre-test scores. Among anesthesia providers (*n* = 32), mean scores increased from 12.7 ± 1.6 to 14.7 ± 0.5 (*t*(31) = 8.2, *p* < 0.0001). Medical doctors (*n* = 23) showed an increase from 12.0 ± 2.1 to 14.7 ± 0.6 (*z* = –3.9, *p* < 0.0001). Nurses (*n* = 69) demonstrated the largest improvement, with mean scores increasing from 10.1 ± 2.6 to 14.4 ± 0.8 (*t*(68) = 15.8, *p* < 0.0001). Smaller groups, including anesthesia students (*n* = 6), dentists (*n* = 1), and nurse assistants (*n* = 3), also showed increases in mean scores; however, statistical testing was not performed due to the limited sample sizes.

Participants trained by independent trainers achieved similar improvements to those trained under direct faculty supervision. Participants taught by faculty trainers (n = 37) demonstrated an increase in mean scores from 10.9 ± 2.6 to 14.6 ± 0.6 (t(36) = 9.5, p < 0.0001). Those trained under faculty supervision (n = 27) showed comparable gains, with mean scores increasing from 10.6 ± 2.6 to 14.4 ± 0.7 (z = –4.5, p < 0.0001). Participants trained by independent trainers (n = 70) also demonstrated significant improvement, with mean scores rising from 11.4 ± 2.4 to 14.6 ± 0.7 (t(69) = 12.1, p < 0.0001). These findings indicate that similar knowledge acquisition was achieved regardless of trainer type. A similar pattern was observed in participants’ performance during the Basic Life Support (BLS) practical skills assessment, where high competency was demonstrated across all training groups.

### Follow-up outcomes (4–6 week survey)

#### Self-reported application of learning in practice.

**Fig 2:**
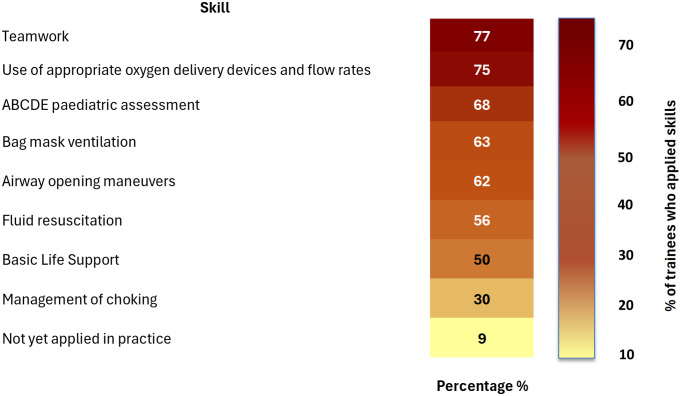
Application of learning following training (4–6-week follow-up).

At 4–6 weeks post-training, 90 participants (67% response rate) completed the follow-up survey. Respondents across all sites reported widespread application of core clinical skills within 4–6 weeks following the training (Fig 2).

Participants reported several barriers to applying their newly acquired skills and knowledge following the StART training. Of the 77 participants who responded to the question on barriers, 43% (*n* = 33) indicated that they did not encounter any significant barriers, however, 57% (*n =* 44) reported experiencing at least one challenge. The most frequently cited barrier was a lack of resources or equipment (57%, *n* = 44). Less commonly reported barriers included insufficient support from colleagues (5%, *n* = 4), resistance to change within the workplace (4%, *n* = 3), and lack of confidence in applying new skills (3%, *n* = 2).

### Trainer survey

All participants from the three countries completed the follow-up survey after the ToT course (N = 14). Responses indicated consistently high levels of agreement regarding both the effectiveness of the ToT program and participants’ confidence in applying the training at their facilities. Across all sites, 78.6% (n = 11) strongly agreed and 21.4% (n = 3) agreed that the balance of ToT activities was effective. Similarly, 78.6% (n = 11) strongly agreed and 21.4% (n = 3) agreed that they felt confident to continue teaching StART Pediatric BLS at their facilities. All respondents (100%, n = 14) strongly agreed that their facility would benefit from ongoing StART Pediatric BLS training.

### Thematic analysis results

Open-ended responses from immediate post-course, 4–6-week follow-up, and ToT surveys were coded and grouped into broader categories using reflexive thematic analysis (Braun & Clarke, 2006). Eight themes were generated: three from the immediate post-course evaluations, three from the 4–6-week follow-up surveys, and two from the ToT trainer feedback. Thematic findings with representative quotations are summarized in Table 4 and described below.

**Table 4:** Summary of thematic findings.

Theme	Summary Description	Illustrative Example (Quote)
**Acquisition of Practical Emergency Care Skills**	Participants gained core pediatric emergency competencies including BLS, choking management, oxygen therapy, and assessment.	“I will be able to identify the casualty who needs CPR and act accordingly because right now I am well equipped with the knowledge and skills.” – Trainee, Uganda
**Confidence and Teamwork in Emergency Response**	Participants described increased confidence in responding to emergencies and valued interprofessional teamwork.	“It will help make better assessment of a paediatric patient who needs urgent intervention. And now I provide a high-quality CPR, bearing in mind teamwork and leadership” – Trainee, Nigeria
**Ongoing Learning and Skill Retention**	Emphasis on continuous learning and refresher training to maintain competency.	“These trainings must be held every year and for every health professional.” – Trainee, Uganda
**Motivation to Apply Skills in Clinical Practice**	Participants applied BLS and oxygen administration skills in real clinical cases post-course.	“We had a paediatric patient who had a cardiac arrest on the operating table... the baby recovered very well.” – Trainee, Nigeria
**Confidence and Teamwork Enabling Action**	Team-based response and increased individual confidence during emergencies.	“We did open airway OPA and ambu... we worked as a team.” – Trainee, Rwanda
**Context-Specific Capacity Building**	Trainers valued the course’s simplicity, relevance, and adaptability to local contexts.	“We will easily manage to teach other hospital staff no matter the cadre… It leads down to less mortality and morbidity.” – Trainer, Uganda

### Immediate post course reflections

Participants described the course as highly practical, highlighting both the acquisition of essential emergency care skills and the broader benefits of teamwork, confidence, and the need for ongoing learning.

#### Theme 1: acquisition of practical emergency care skills.

Participants consistently highlighted gaining core competencies in pediatric emergency care, including BLS, choking management, oxygen therapy, and patient assessment.

“I will be able to identify the casualty who needs CPR and act accordingly because right now I am well equipped with the knowledge and skills.” (Trainee Uganda)

“Percentage of oxygen delivered by different oxygen giving devices.” (Trainee Nigeria)

#### Theme 2: confidence and teamwork in emergency response.

Participants described increased confidence in responding to emergencies and valued working collaboratively with colleagues from different professional backgrounds. A trainee from Uganda stated, “This course will has helped me to act in more confident and skilled way during emergencies and daily routines”. While another commented, “It will help make better assessment of a pediatric patient who needs urgent intervention. And now I provide a high-quality CPR, bearing in mind teamwork and leadership.” (Trainee Nigeria). On a similar note “The importance of teamwork at achieving better result.” was raised by a trainee from Rwanda.

#### Theme 3: ongoing learning and skill retention.

Participants highlighted the need for continued practice and refresher training to maintain and build on newly acquired skills. “These trainings must be held every year and for every health professional.” (Trainee Uganda). Another stated,

“A refresher course will be highly appreciated to keep health workers abreast of current resuscitation skills and practice.” (Trainee Nigeria)

### Follow-up reflections (4–6 weeks post-course)

By 4–6 weeks after the course, participants reported applying the skills in clinical settings, reinforcing the practical value of training.

#### Theme 1: motivation to apply skills in clinical practice.

Participants reported real-world application of skills such as BLS, oxygen administration, and patient assessment. Several shared vivid accounts:

“I had a conscious 9-year-old with signs of increased work of breathing and SPO2 87% on nasal prongs. We changed her to a non-rebreather mask and her SPO2 went up to 98% as we dealt with the cause of respiratory distress.” (Trainee, Uganda)

“We had a pediatric patient who had a cardiac arrest on the operating table, and we applied exactly what we learned during StART, and to the Glory of God the baby recovered very well.” (Trainee, Nigeria)

#### Theme 2: active engagement in learning and practice.

Trainees reported that the course strengthened their understanding of emergency care and helped them make clearer clinical decisions. One reflected, *“I am now more conscious and deliberate about my primary and secondary survey and that has really helped in the Emergency unit.”* (Trainee, Nigeria) Another added, “The way I used to give oxygen has been changed… I learnt how to react emergencely [sic], the steps to follow without wasting time, and how to use bag-mask ventilation.” (Trainee, Nigeria)

#### Theme 3: confidence and teamwork enabling action.

Participants described acting with greater confidence, but this was frequently framed in terms of working collaboratively with colleagues. Team-based resuscitation was seen as both a source of confidence and a requirement for effective practice:

*“The patient was desaturating post extubation, we did open airway OPA and ambu, in a short time the patient’s condition improved and later transferred to ward. We worked as a team.”* (Trainee, Rwanda)

Confidence also extended to individual roles within the team, particularly when responding under pressure:

*“Am more confident when called to respond to emergencies. My knowledge has increased and I now know the rationale of my interventions.”* (Trainee, Uganda)

### Trainer perspectives on the ToT Model

New trainers emphasized the relevance of StART for their local context, its potential for scale-up, and its role in building sustainable capacity across professions.

#### Theme 1: context-specific capacity building.

Trainers viewed StART as well suited to low-resource African hospitals, highlighting its practical, scenario-based approach and ease of scale-up. As one Ugandan trainer explained:

“StART Pediatric BLS is designed to be delivered in a simple way and it is a one-day course. We will easily manage to teach other hospital staff no matter the cadre… It leads down to less mortality and morbidity.” (Trainer, Uganda)

#### Theme 2: sustainability and training reach.

The ToT model was widely valued for its potential to embed resuscitation skills locally. A Nigerian trainer reflected,

*This course definitely will improve the level of knowledge and practice of healthcare personnel in my facility. Also, it will at the end of the day improve the level of care in terms of emergency at pediatric care-giving points.*

Another trainer simply stated, “This training is vitally important in our setting, all workers need it and the time to keep practicing so we are ready for an emergency” (Trainer, Uganda).

### Summary of mixed-methods findings

Taken together, the quantitative and qualitative findings show clear improvements in knowledge, practical skills, and confidence, with participant-reported evidence that learning was being applied in their daily work. The consistency of results across different professional groups and countries suggests that the StART model is adaptable and relevant in a range of hospital settings.

## Discussion

### Feasibility and acceptability

Findings from participants and facilitators indicate that the StART program was highly feasible to deliver and broadly acceptable across all participating sites. This mixed-methods evaluation demonstrated that the one-day StART Pediatric Resuscitation course is both practical and contextually appropriate for diverse African hospital settings. Participants consistently described the training as relevant, hands-on, and aligned with the realities of their clinical environments. The concise one-day structure was especially valued, as it allowed busy staff to attend without major disruption to service delivery, a critical factor in workforce-limited settings and one echoed in recent evaluations of simulation-based training feasibility in LMICs. [21]

Prior to StART, most participants reported no recent or prior structured pediatric resuscitation training, highlighting a substantial skills gap. This aligns with more recent research showing that nurses and clinicians in LMICs often perform emergency care responsibilities without adequate formal training, contributing to preventable morbidity and mortality [22]. The positive endorsement of StART from all cadres therefore reflects both the relevance of the content and the urgent workforce needs it addresses.

Although knowledge gains varied between professions, with nurses demonstrating the largest relative improvements, there were no differences among professional groups in their endorsement of the training. All cadres, including doctors, anesthetists, and nurses, described the course as accessible, practical, and applicable to their roles. This is consistent with contemporary evidence demonstrating that interprofessional simulation-based education enhances teamwork, communication, and coordination in emergency care [23,24]. Interprofessional training in hierarchical healthcare settings can present challenges, including differences in confidence, communication patterns, and willingness to speak up among professional groups. While this was not formally measured in the present study, the course was intentionally designed to promote inclusive participation across disciplines. Small group learning, mixed professional grouping, and hands-on simulation were used to encourage interaction and shared decision-making. Informally, participants demonstrated active engagement across professional roles, suggesting that these approaches may help support psychological safety and collaborative learning in multidisciplinary settings. Trainers and participants emphasized that the scenario-based design and practical skills stations supported strong engagement and facilitated plans for local peer-to-peer teaching. As one Ugandan trainer noted, “StART Pediatric BLS is designed to be delivered in a simple way and it is a one-day course. We will easily manage to teach other hospital staff no matter the cadre.” This sentiment is supported by recent work highlighting that concise, context-appropriate simulation formats improve local ownership and participation across cadres [25].

Together, these findings reinforce that StART is feasible to deliver, highly acceptable to participants, and appropriate for multidisciplinary teams across resource-limited hospital settings. As a pediatric resuscitation training program developed for sub-Saharan African hospitals, StART was designed with scalability in mind, including the potential for adaptation across languages and settings. This adaptability strengthens its feasibility for wider regional implementation.

### Effectiveness and application to practice

StART effectively strengthened participants’ knowledge and their reported ability to apply pediatric resuscitation skills in clinical practice. Across all sites, participants showed improvements across key components of pediatric emergency care, including airway support, oxygen therapy, and basic life support. All participants met BLS competency standards at course completion. Participants reported increased confidence and a greater perceived readiness to use these skills in real clinical situations. In addition to statistically significant improvements, the magnitude of learning gains was substantial, with a very large effect size observed between pre- and post-training knowledge scores (Cohen’s d = 1.5). Given the intensive, short-course nature of the training and participants’ limited prior formal exposure, the large observed effect size highlights the practical educational significance of the intervention and supports its effectiveness as a focused, context-specific pediatric resuscitation training program. These findings align with recent literature showing that structured pediatric emergency training programs in LMICs can produce meaningful improvements in clinical performance [26,27].

Qualitative reflections from the four- to six-week post-training period provided evidence of participant-reported application of learning in clinical practice. Participants reported applying StART skills during acute presentations, including respiratory distress and cardiac arrest, and described adjusting interventions based on patient response. Themes such as “Motivation to Apply Skills in Clinical Practice” and “Active Engagement in Learning and Practice Improvement” reflected participants’ motivation to integrate learning into care delivery. Recent studies similarly highlight that self-efficacy gained through simulation-based and structured resuscitation programs may support sustained behavior change and performance during emergencies [22,28].

Increased confidence and clearer role allocation within teams were reported by participants, who described greater readiness to respond to emergencies. These reflections align with evidence from interprofessional training, which has been shown to improve teamwork and communication processes that are key enablers of safe, coordinated resuscitation performance [21,29].

The Capability, Opportunity, Motivation-Behavior (COM-B) model provides a useful lens for interpreting these findings.[30] Participants demonstrated capability through measured improvements in knowledge and skills, motivation through strong engagement and growing confidence, and opportunity through the course’s team-based delivery and realistic, context-specific materials. Nonetheless, participants also identified systemic barriers such as limited equipment availability and variable institutional support. These challenges are documented in the LMIC emergency care literature and contribute to persistent performance gaps despite training [31]. This underscores the need for educational interventions such as StART to be complemented by supportive health system structures to fully enable sustained practice change [32]. Future iterations of the program could build on these findings by addressing some of the practical barriers identified, such as equipment availability, alongside continued support for team-based practice. Although patient-level outcomes were not measured in this study, improvements in early recognition and resuscitation skills may be clinically relevant in LMIC settings where delays in identifying and responding to pediatric deterioration contribute to preventable morbidity and mortality. In Malawi and Mozambique, structured pediatric emergency triage and treatment approaches, including ETAT-based training, have been associated with reductions in pediatric inpatient or emergency department mortality [33,34]. These findings support the potential relevance of StART as part of broader efforts to strengthen pediatric emergency care, while recognizing that this evaluation cannot establish causal links with patient outcomes.

Together, the quantitative and qualitative evidence indicate that StART is an effective, implementable, and clinically relevant training model with potential to strengthen pediatric emergency care across resource-constrained settings.

### Sustainability and ToT model

The ToT component was essential in promoting local ownership and long-term sustainability across all three implementation sites. Participants consistently emphasized their need for regular refresher opportunities, reflecting established evidence that resuscitation knowledge and psychomotor skills begin to deteriorate within months without reinforcement [35–37]. The StART ToT model is designed to address this challenge by preparing selected participants to deliver the course within their own institutions, thereby strengthening continuity and reducing dependence on external facilitators.

During the two-day ToT program, new trainers received structured instruction in facilitation, learner assessment, debriefing techniques, and mentorship skills before being signed off to teach independently. Follow-up surveys demonstrated strong acceptability and readiness to assume training roles. Seventy-nine percent of trainers strongly agreed that the balance of ToT activities effectively prepared them for course delivery, and all reported confidence in their ability to continue teaching within their facilities. A trainer from Uganda emphasized the importance of ongoing practice, noting that the training is vital in their setting and that health workers require dedicated time to maintain readiness for emergencies. This perspective aligns with prior research showing that contextualized and practice-oriented approaches enhance engagement, relevance, and sustained performance among health workers in low-resource settings [38].

The ToT model is further supported by global evidence showing that locally led instructional approaches strengthen workforce performance and promote sustainable capacity building in resource-constrained health systems [39]. By developing a cadre of trainers embedded within health facilities, the StART program facilitates iterative practice, local adaptation, and the integration of pediatric assessment and early resuscitation skills into routine clinical workflows. These features reflect key principles identified in implementation science frameworks, which emphasize contextual adaptation, institutionalization, and local ownership as central to achieving durable program uptake [40,41].

Although follow-up data captured participant-reported early application of learning, formal reassessment of knowledge and skills at 4–6 weeks was not undertaken. This reflected the design of the pilot implementation, which focused on initial course delivery and follow-up through participant-reported outcomes. While this approach supported feasibility across diverse sites, it limited the ability to assess short-term knowledge retention. Future evaluations should incorporate repeat assessment to better understand retention of learning over time.

Taken together, the findings indicate that the StART course is both educationally effective and operationally feasible. Its context-specific design, multidisciplinary approach, and embedded sustainability mechanisms position it as a practical and scalable model for strengthening pediatric emergency care capacity across African hospitals.

### Strengths and Limitations

A major strength of this evaluation is its mixed-methods design, which allowed for triangulation of quantitative test results with qualitative feedback to generate a comprehensive understanding of the course’s impact. Implementing StART across three countries enhanced external validity and demonstrated the adaptability of the training model across diverse health system contexts. The inclusion of multidisciplinary participants further supports the program’s relevance and feasibility for varied clinical teams.

Several limitations should be considered when interpreting these findings. First, the study used convenience sampling of three hospital sites, which may limit the representativeness of the sample and the generalizability of results to other settings. Although implementation across multiple countries supports broader relevance, differences in institutional resources, staffing, and organizational support may influence how the program is delivered and sustained in other contexts.

Second, while follow-up data provided insight into participant-reported early application of learning, the response rate at 4–6 weeks was 67%, and those who chose to respond may not fully represent the entire participant group. It is possible that individuals who were more engaged with the training or more confident in applying their skills were more likely to complete the survey. In addition, the relatively short follow-up period limited the ability to assess longer-term knowledge and skills retention or sustained changes in clinical practice.

Third, improvements in knowledge, confidence, and reported clinical practice were based primarily on participant self-report and assessment outcomes within the training environment, rather than direct observation of clinical performance. While these findings provide important insight into perceived impact and competency acquisition, they may not fully reflect changes in clinical practice or patient outcomes. Although standardized checklists, predefined competency criteria, trainer preparation, and faculty oversight supported consistency in the practical BLS skills assessment, formal inter-rater reliability testing was not undertaken.

In Rwanda, opportunities for follow-on training were limited during the study period. This appeared to reflect a combination of smaller facility size and lower staff turnover, which reduced the immediate need for additional training cycles. The relatively small sample size at the Rwanda site and the limited opportunity for follow-on training also mean that it is difficult to draw conclusions about how the model would scale in smaller institutions.

Future research should include longer-term follow-up, direct observation of clinical practice, and evaluation of patient-level outcomes to further assess the impact and sustainability of the program.

## Conclusions and next steps

This multi-country pilot provides early evidence that the StART Pediatric Resuscitation course is both feasible to implement and valuable for strengthening pediatric emergency care capacity in low-resource hospital settings. The program addressed a clearly identified training gap among experienced clinicians, improved participants’ knowledge and practical skills, supported participant-reported improvements in teamwork, and established a foundation for ongoing capacity-building through the ToT model. With continued refinement and sustained implementation, StART has the potential to contribute meaningfully to improved pediatric emergency preparedness across African health systems.

Future work should expand implementation to additional sites and incorporate longer-term follow-up to assess knowledge retention, skill maintenance, and the durability of participant-reported behavior change. Understanding how StART can be integrated into existing institutional, regional, or national training systems will be essential for scaling, as will evaluating cost-effectiveness and resource requirements. These next steps will be important for determining the program’s long-term sustainability and its potential to support regional improvements in pediatric emergency care outcomes.

## Supporting information

S1 Data Pilot Qualitative and Quantitative Data. (XLSX)

S1 Text Pre-course Test. (PDF)

S2 Text Post-Course Test. (PDF)

S3 Text BLS Skills Test. (PDF)

S4 Text Trainee Evaluation Form. (PDF)

S5 Text TOT Trainer Evaluation Form. (PDF)

S6 Text Follow Up Form. (PDF)

S1 Checklist Inclusivity-in-global-research-questionnaire. (DOCX)
