# Supplementary material for: Pediatric assessment and early resuscitation capacity building: Multi-country implementation of the StART Training-of-Trainers Model in Rwanda, Uganda, and Nigeria
Source: PLOS Glob Public Health. 2026 Aug 14;6(8):e0006137. doi: 10.1371/journal.pgph.0006137 (PMC13475823; doi:10.1371/journal.pgph.0006137)
Supplement: Supplementary file 1 [file pgph.0006137.s002.pdf]

Pre-Course StART Exam

Q1. What is the most common cause of cardiac arrest in infants and children?
a) Primary cardiac arrhythmia
b) Respiratory failure leading to hypoxia
c) Brain injury

Q2. A child is breathing fast and has chest retractions but is alert and responsive. What is the most appropriate first action?
a) Start chest compressions
b) Give intravenous fluids
c) Give oxygen

Q3. When checking breathing in an unresponsive infant or child, how long should you take?
a) Less than 3 seconds
b) No more than 10 seconds
c) Exactly 5 seconds

Q4. During chest compressions for an infant or child, how deep should you compress the chest?
a) 1/2 of the chest depth
b) 1/3 of the chest depth
c) Compress lightly to avoid breaking ribs

Q5. What is the correct ratio of chest compressions to breaths in an infant or child victim?
a) 15 compressions: 2 breaths
b) 20 compressions: 5 breaths
c) 20 compressions: 2 breaths

Q6. If an infant is choking and conscious, what should you do first?
a) Start CPR
b) Begin with 5 chest thrusts
c) Begin with 5 back blows

Q7. You find a child who is unresponsive and not breathing; after shouting for help, what should you do next?
a) Open the airway and give 5 rescue breaths
b) Start with 15 chest compressions
c) Give high flow oxygen with a simple face mask

Q8. Which oxygen delivery device provides high-flow oxygen (>80%) to a 2-year-old child?
a) Nasal cannula at 4 litres per minute (LPM)
b) Non-rebreather face mask at 15 litres per minute (LPM)
c) Simple face mask at 15 LPM

Q9. A 6-month-old infant has an AVPU of P
Respiratory rate is 7, heart rate of 75, SPO2 of 81%, the infant is cyanosed
The infant shows signs of:
a) Respiratory distress
b) Cardiac arrest
c) Respiratory failure

Q10) What is the immediate action for the infant in Q9?
a) Give oxygen with a simple face mask 10 litres per minute (LPM)
b) Give oxygen via nasal cannula at 4 litres per minute (LPM)
c) Shout for help and start bag-mask ventilation

Q11. During a respiratory assessment of an infant, you hear a high-pitched sound during inspiration. What is this likely to be?
a) Stridor
b) Wheezing
c) Snoring

Q12) A 4-year-old child has had diarrhoea for 4 days. The child is lethargic, has a RR is 40, HR is 130, and capillary refill time is 4 seconds. What action will you take?
a) Give normal saline 500mls over 4 hours
b) Give oxygen via nasal cannula at 4 LPM
c) Give a bolus of 10-20 mL per kg of Ringer's lactate.

Q13. Which sign shows increased work of breathing in a child?
a) Chest retractions
b) Reduced level of responsiveness
c) Delayed capillary refill time (CRT)

Q14. When giving oxygen to a child, what is the target SPO_2 range?
a) 100%
b) 90-94%
c) 94-98%

Q15. A child has a history of vomiting and fever. Which signs suggest hypovolemic shock?
a) Skin rash, pyrexia and high heart rate
b) Rapid breathing, high heart rate and capillary refill time of 5 seconds
c) lethargy, normal heart rate and rapid breathing
