# Supplementary material for: Pediatric assessment and early resuscitation capacity building: Multi-country implementation of the StART Training-of-Trainers Model in Rwanda, Uganda, and Nigeria
Source: PLOS Glob Public Health. 2026 Aug 14;6(8):e0006137. doi: 10.1371/journal.pgph.0006137 (PMC13475823; doi:10.1371/journal.pgph.0006137)
Supplement: Supplementary file 2 [file pgph.0006137.s003.pdf]

**Post-course Exam Start Paediatric BLS**

Q1. When administering oxygen therapy to a child, what is the target SPO2 range?
a) 94-98%
b) 92-94%
c) 92% or above

Q2. During chest compressions for an infant or child, what is the correct compression depth?
a) 1/4 of the chest depth
b) As deep as possible
c) 1/3 of the chest depth

Q3. A 9-month-old infant presents with an AVPU score of P, a respiratory rate of 8, heart rate of 65, SPO2 of 80%, and cyanosis. Your assessment indicates the child is in:
a) Respiratory failure
b) Respiratory distress
c) Hyperglycaemia

Q4. In response to the scenario in Q3, what immediate intervention is required?
a) Give oxygen via nasal cannula at 4 LPM
b) Give the child water to drink
c) Call for help, initiate bag-mask ventilation

Q5. A child with a history of vomiting and fever displays tachycardia, weak peripheral pulses, and a capillary refill time of 5 seconds. These signs suggest:
a) Respiratory distress
b) Hypovolemic shock
c) Allergic reaction

Q6. In BLS for an infant or child what is the correct compression-to-ventilation ratio?
a) 20:2
b) 30:1
c) 15:2

Q7. When assessing an unresponsive infant or child, how long should you take to check if they are breathing normally?
a) 15 seconds
b) No more than 10 seconds
c) 1-2 seconds

Q8. Which of the following is a sign of increased work of breathing in a child?
a) Elevated temperature
b) Chest retractions
c) Raised blood glucose

Q9. You find an unresponsive child not breathing; after shouting for help, what should you do next?
a) Give 15 compressions
b) Give 15 back blows
c) Open the airway and give 5 rescue breaths

Q10. A child with severe respiratory distress is receiving oxygen via nasal cannula at 4 LPM. The child becomes drowsy and the respiratory rate drops. What is your next immediate action?
a) Increase nasal cannula flow to 6 LPM
b) Call for help and begin bag-mask ventilation
c) Check blood glucose

Q11. A 3-year-old child has a respiratory rate of 51, which oxygen delivery method provides high-flow oxygen (>80%)?
a) Non-rebreather face mask at 15 LPM
b) Nasal cannula at 6 LPM
c) Simple face mask at 12 LPM

Q12. During a respiratory assessment of an infant, you observe a high-pitched sound during inspiration. What is this likely to be?
a) Asthma
b) Normal breathing
c) Stridor

Q13. What is the most common cause of cardiac arrest in infants and children?
a) Asthma
b) Accidents
c) Respiratory failure

Q14. A 5-year-old boy with a 2-day history of diarrhoea is lethargic, has a respiratory rate of 35, heart rate of 120, capillary refill time of 4 seconds. What action should you take?
a) Give 250ml fluid over 2 hours
b) Review the child in 1 hour and give reassurance
c) Give 10-20 mL per kg of normal saline as a bolus

Q15. If an infant is conscious and choking, what should be your initial action?
a) Leave the child and get help
b) Start CPR
c) Administer back blows
