# Supplementary material for: Pediatric assessment and early resuscitation capacity building: Multi-country implementation of the StART Training-of-Trainers Model in Rwanda, Uganda, and Nigeria
Source: PLOS Glob Public Health. 2026 Aug 14;6(8):e0006137. doi: 10.1371/journal.pgph.0006137 (PMC13475823; doi:10.1371/journal.pgph.0006137)
Supplement: Supplementary file 3 [file pgph.0006137.s004.pdf]

Basic Life Support Practical Testing

Participant Name:

Infant CPR Testing

Infant Scenario: You are working on a pediatric ward, a mother brings you her child who has suddenly become floppy child, saying ‘help, my baby’ the child is 3 months old.

Demonstrate what you will do next

☐ Checks for responsiveness

☐ Shouts for help

☐ Opens airway

☐ Checks breathing ☐ Checks for signs of life or a brachial pulse

☐ Gives 5 rescue breaths ☐ Each breath over 1 second ☐ Chest rise observed

If no signs of life

☐ Gives 15 chest compressions

☐ Correct placement of fingers or hands

☐ Correct depth 1/3rd chest

☐ Correct rate (120 per minute)

☐ Allows chest recoil

☐ Gives 2 breaths

Help arrives, to join resuscitation (2 rescuers)

☐ Continues 15 compressions : 2 breaths

☐ Gives clear communication

☐ Minimises interruptions, resumes chest compressions in less than 10 seconds

Trainer Name:

Outcome

1 Station Pass

2 Requires practice Station Pass

Child CPR

Child Scenario: You are working on the pediatric surgical ward, you go the bedside of a child and find they are unresponsive, the child is 5 years old

Demonstrate what you will do next

☐ Checks for responsiveness

☐ Shouts for help

☐ Opens airway

☐ Checks breathing ☐ Checks for signs of life or a carotid pulse

☐ Gives 5 rescue breaths ☐ Each breath over 1 second ☐ Chest rise observed

If no signs of life

☐ Gives 15 chest compressions

☐ Correct placement of fingers or hands

☐ Correct depth 1/3rd chest

☐ Correct rate (120 per minute)

☐ Allows chest recoil

☐ Gives 2 breaths

Help arrives, to join resuscitation (2 rescuers)

☐ Continues 15 compressions : 2 breaths

☐ Gives clear communication

☐ Minimises interruptions, resumes chest compressions in less than 10 seconds

Trainer Name:

Outcome

1 Station Pass

2 Requires practice Station Pass
