# Supplementary material for: Pediatric assessment and early resuscitation capacity building: Multi-country implementation of the StART Training-of-Trainers Model in Rwanda, Uganda, and Nigeria
Source: PLOS Glob Public Health. 2026 Aug 14;6(8):e0006137. doi: 10.1371/journal.pgph.0006137 (PMC13475823; doi:10.1371/journal.pgph.0006137)
Supplement: Supplementary file 4 [file pgph.0006137.s005.pdf]

StART Trainee Evaluation Pilot

Thank you for attending the StART Pediatric BLS pilot.

We value your feedback as it helps us evaluate the course and improve future training.

Completing this form is voluntary. All responses are confidential and will be summarized anonymously.

By continuing, you are agreeing to take part in this evaluation.

Thank you for taking the time to share your thoughts.

* Indicates required question

1. Please select your profession from the list below * Dropdown

Mark only one oval.

- Nurse
- Anesthetist
- Anesthesiologist
- Doctor
- Surgeon
- Paeditrician
- Health care assistant
- Other

2. How many years have you practised in your profession? * Dropdown

Mark only one oval.

- ◯ <1
- ◯ 1-2
- ◯ 3-5
- ◯ 6-10
- ◯ More than 10

3. Before this training, have you previously attended training on: *

Mark only one oval per row.

	Yes	No
**Pediatric ABCDE Assessment**	◯	◯
**Pediatric Intervention (airway management, oxygen delivery options, fluid resuscitation)**	◯	◯
**Pediatric BLS**	◯	◯

4. If you answered yes in question 3 to previously attending BLS training, was the training more than 2 years ago? Dropdown

Mark only one oval.

- ◯ Yes
- ◯ No

5. This course has improved my professional expertise * Dropdown

Mark only one oval.

- Significantly
- Somewhat
- Not at all

6. Would you recommend this course to others Dropdown

Mark only one oval.

- Yes
- No
- Maybe

7. Following this training, do you feel more competent to assess and intervene if a child is critically ill in your workplace Dropdown

Mark only one oval.

- Yes
- No
- Maybe

8. Rate the course under the following headings: *

Mark only one oval per row.

	Excellent	Very good	Good	Fair	Poor
**Presentations**	◯	◯	◯	◯	◯
**Pace of course**	◯	◯	◯	◯	◯
**Learning methods**	◯	◯	◯	◯	◯
**Trainers**	◯	◯	◯	◯	◯
**Course material**	◯	◯	◯	◯	◯

9. What was the most useful thing you learnt today *

____

10. How will this course help you in your current role *

____

11. Who do you think this course is suitable for? * Dropdown

Mark only one oval.

- ◯ All healthcare workers who assess and care for pediatric patients
- ◯ Mostly nurses
- ◯ Anaesthesia providers only
- ◯ Medical doctors and surgeons only
- ◯ Other

12. What if anything, would you change on this course?

____

13. The length of the training was? * Dropdown

Mark only one oval.

- ( ) Just right
- ( ) Too long
- ( ) Too short

14. If you would like to make any further comments about this training, please do so below

____

This content is neither created nor endorsed by Google.

Google Forms
