# Supplementary material for: Pediatric assessment and early resuscitation capacity building: Multi-country implementation of the StART Training-of-Trainers Model in Rwanda, Uganda, and Nigeria
Source: PLOS Glob Public Health. 2026 Aug 14;6(8):e0006137. doi: 10.1371/journal.pgph.0006137 (PMC13475823; doi:10.1371/journal.pgph.0006137)
Supplement: Supplementary file 5 [file pgph.0006137.s006.pdf]

Pilot StART ToT Trainer Evaluation

Thank you for attending the Pilot StART Pediatric BLS Training of Trainers (ToT)
We value your feedback as it helps us evaluate the course and improve future training.

Completing this form is voluntary. All responses are confidential and will be summarized anonymously.

By continuing, you are agreeing to take part in this evaluation.

Thank you for taking the time to share your thoughts on your ToT experience.

* Indicates required question

1. At the end of days 1-3 (ToT programme) I felt ready to teach StART Pediatric BLS? * Dropdown

   Mark only one oval.

   - Strongly Agree
   - Agree
   - Neutral
   - Disagree
   - Strongly Disagree

2. The balance of ToT activities (course participation, practice presentations & activities, discussion and presentations) was effective? * Dropdown

   Mark only one oval.

   - Strongly Agree
   - Agree
   - Neutral
   - Disagree
   - Strongly Disagree

3. If you disagree or strongly disagree with question 2 please comment here how it could be improved:

____
____
____
____
____

4. I felt supported by faculty teaching StART Pediatric BLS (day 4)? * Dropdown

Mark only one oval.

- Strongly Agree
- Agree
- Neutral
- Disagree
- Strongly Disagree

5. The length of the ToT training was? * Dropdown

Mark only one oval.

- Just right
- Too long
- Too short

6. I am confident to continue teaching StART Pediatric BLS at my facility on an ongoing basis — Dropdown

Mark only one oval.

- Strongly agree
- Agree
- Neutral
- Disagree
- Strongly Disagree

7. My facility will benefit from ongoing StART Pediatric BLS training * — Dropdown

Mark only one oval.

- Strongly agree
- Agree
- Neutral
- Disagree
- Strongly Disagree

8. If you answered Agree or Strongly agree for question 7, state how it will benefit

____

9. What if anything would you change in the ToT

____

10. If you would like to make any further comments about the ToT training, please do so below

This content is neither created nor endorsed by Google.

Google Forms
