# Supplementary material for: Pediatric assessment and early resuscitation capacity building: Multi-country implementation of the StART Training-of-Trainers Model in Rwanda, Uganda, and Nigeria
Source: PLOS Glob Public Health. 2026 Aug 14;6(8):e0006137. doi: 10.1371/journal.pgph.0006137 (PMC13475823; doi:10.1371/journal.pgph.0006137)
Supplement: Supplementary file 6 [file pgph.0006137.s007.pdf]

StART Training Follow-up Questionnaire

Thank you for attending the recent StART Pediatric BLS pilot course.

We are interested in your experience since the training, including whether and how you have been able to use what you learned in practice.

Your honest feedback, both positive and critical, helps us evaluate the course and improve future training. We appreciate you taking the time to complete this brief survey.

Participation is voluntary. All responses are confidential and will be summarized anonymously.

By continuing with this form, you are agreeing to take part in this evaluation.

Thank you again for sharing your experiences.

* Indicates required question

1. 1 Where did you attend StART? *

____

2. 2 Since attending StART has your care of of pediatric patients changed in any way?

Mark only one oval.

- ◯ Yes
- ◯ No

3. 2a If you answered yes to question 2, briefly state what you now do differently

____

4. 3 Which skills or knowledge you learnt on StART have you been able to apply in your practice? (select all that apply)

Check all that apply.

- [ ] ABCDE pediatric assessment
- [ ] Use of appropriate oxygen delivery devices and flow rates
- [ ] Airway opening manoeuvres
- [ ] Fluid resusciation
- [ ] Bag mask ventilation
- [ ] Basic Life support
- [ ] Management of choking
- [ ] Teamwork
- [ ] No skills or knowledge have been applied in practice yet

5. 4 Since attending StART can you think of a specific occasion/s when you applied what you learnt in practice *

Mark only one oval.

- ( ) Yes
- ( ) No

6. 4a If you answered yes to question 4 please briefly describe the situation, your intervention and the outcome, if known (do not state any details that might identify the patient)

7. 5 Since attending StART have you noticed any improvement any of the following * in your workplace

Check all that apply.

- [ ] Pediatric assessment
- [ ] Intervention (airway support, oxygen delivery, fluid resuscitation, management of choking)
- [ ] Basic Life Support (BLS)
- [ ] No improvement noted yet

8. 6 What barriers, if any, have you encountered when trying to implement what you learned in the course? (Select all that apply)

Check all that apply.

- [ ] Lack of equipment or resources
- [ ] Insufficient support from colleagues
- [ ] Lack of confidence in applying learning or techniques
- [ ] Resistance to change from team members
- [ ] No significant barriers encountered
- [ ] Other: ____________________

9. 6a If you noted barrier/s above, please state what could be done to overcome these barriers

10. 7 Since StART was delivered at your facility have you noted improvements in any of the following items of emergency preparedness? (tick any noted)

Check all that apply.

- [ ] Availability of oxygen pediatric delivery devices (including non-rebreather face mask)
- [ ] Availability of oxygen
- [ ] Availability of pediatric airways
- [ ] Availability of pediatric bag mask device (AMBU)
- [ ] Availability of suction
- [ ] Availability of an emergency tray or trolley
- [ ] Means to call for or access help in an emergency
- [ ] No improvement noted

11. 8 What was the most important thing you learnt during StART *

____

12. 9 Do you have any additional comments or suggestions regarding the course or its application in your practice?

____

This content is neither created nor endorsed by Google.

Google Forms
