# Supplementary material for: Pediatric assessment and early resuscitation capacity building: Multi-country implementation of the StART Training-of-Trainers Model in Rwanda, Uganda, and Nigeria
Source: PLOS Glob Public Health. 2026 Aug 14;6(8):e0006137. doi: 10.1371/journal.pgph.0006137 (PMC13475823; doi:10.1371/journal.pgph.0006137)
Supplement: Supplementary file 9 [file pgph.0006137.s011.docx]

**Manuscript Title:**
*Pediatric assessment and early resuscitation capacity building: Implementation of the StART Training-of-Trainers Model in Rwanda, Uganda, and Nigeria*

**Reviewer #1**

Thank you for your constructive feedback. We appreciate your positive comments on the relevance of this work and have addressed all points as outlined below.

**Comment:** Expansion of intervention description (equipment, venue, ToT selection)

**Response:** Thank you for this suggestion. We have expanded the description of the intervention to provide additional detail on equipment requirements, training venue, and selection of Training-of-Trainers (ToT) participants. This now includes clarification on the use of locally available equipment, hospital-based delivery of training, and the selection criteria used by the hospital leadership for ToT candidates.

**Comment:** Retention testing at 4–6 weeks

**Response:** We agree this is an important point. We have added a statement in the Discussion to clarify that repeat knowledge and skills assessment at 4–6 weeks was not undertaken as part of the pilot design and have acknowledged this as a limitation. We also highlight the importance of incorporating repeat assessment in future evaluations.

**Comment:** Barriers and hierarchy in multidisciplinary training

**Response:** Thank you for highlighting this. We have added a paragraph to the Discussion acknowledging the potential influence of professional hierarchies on participation in multidisciplinary training. We also describe how the course design, including small group learning, mixed professional groups, and simulation-based activities, aimed to support inclusive participation and collaborative learning.

**Comment:** Rwanda site- lack of follow-on training

**Response:** We have expanded the Discussion to clarify that limited follow-on training in Rwanda appeared to reflect contextual factors, including smaller facility size and lower staff turnover, which reduced the immediate need for additional training cycles. This has been included to highlight how local context may influence implementation and scale-up.

**Minor Comment:** Definition of anaesthesia provider

**Response:** We have clarified the definition of anaesthesia providers in the Methods section to reflect the range of clinicians involved, including both physician and non-physician providers such as nurse anesthetists

**Minor Comment:** Missing Figure 2

**Response:** Thank you for noting this. Figure 2 has now been included and appropriately referenced in the revised manuscript.

**Reviewer #2**

Thank you for your constructive feedback and for recognising the relevance of this work. We have addressed each of your points below.

**Comment:** Effect size inconsistency

**Response:** Thank you for identifying this. We have reviewed and corrected the reported effect size to ensure consistency across the manuscript.

**Comment:** Follow-up data, response bias, and generalisability

**Response:** We have strengthened the limitations section to address this point. This now includes acknowledgement of the 67% follow-up response rate and the possibility of response bias, as well as the implications of the short follow-up period for assessing sustained behaviour change and knowledge retention. We have also clarified the implications of convenience sampling for generalisability.

**Comment:** Skills assessment (100% pass rate)

**Response:** We have expanded the Methods section to clarify the structure of the skills assessment, including the use of a structured checklist, defined competency thresholds, and assessor orientation to support consistency. We also clarify that assessments were conducted by course trainers and were not blinded, with oversight and mentorship provided during initial delivery. The Results wording has been revised to reflect that participants met competency standards rather than “passed.”

**Comment:** Link to patient outcomes

**Response:** We have added a statement in the Discussion to contextualise how improvements in knowledge and early resuscitation skills may translate into clinical outcomes, while noting that patient-level outcomes were not measured in this study.

**Comment:** Use of the COM-B framework and implications for future programme development **Response:** Thank you for this suggestion. We have added a sentence to the Discussion outlining how future iterations of the program could address key barriers identified, including equipment availability and support for team-based practice.

**Comment:** Subgroup analyses and statistical power

**Response:** We have added a statement to the limitations section noting that subgroup analyses were exploratory in nature and that the study was not designed to detect differences between groups.

**Comment:** MCQ equivalence (“parallel forms”)

**Response:** We have clarified the description of the MCQ assessments in the Methods section, noting that the pre- and post-tests were developed as parallel forms and that comparability was supported through pilot testing, with similar mean scores observed across both versions.

**Minor Comment:** Abstract clarity

**Response:** We have revised the abstract to include the number of Training-of-Trainers participants (n = 14) and the follow-up response rate (67%), and to align wording regarding competency-based assessment.

**Minor Comment:** Tables and typographical errors

**Response:** We have reviewed all tables and text to ensure internal consistency and corrected minor errors and formatting issues.
