# Supplementary material for: Pediatric assessment and early resuscitation capacity building: Multi-country implementation of the StART Training-of-Trainers Model in Rwanda, Uganda, and Nigeria
Source: PLOS Glob Public Health. 2026 Aug 14;6(8):e0006137. doi: 10.1371/journal.pgph.0006137 (PMC13475823; doi:10.1371/journal.pgph.0006137)
Supplement: Supplementary file 10 [file pgph.0006137.s009.docx]

Thank you for the opportunity to review the submission for PLOS Global Public Health:

“Pediatric assessment and early resuscitation capacity building: Implementation of the

StART Training-of-Trainers Model in Rwanda, Uganda, and Nigeria”

Recommendation: **accept with minor revisions**

This valuable paper outlines the process of development, implementation and assessment of impact of a structured one-day health education intervention training for LMICs that integrates pediatric assessment, early intervention, and high-quality resuscitation skills; developed in response to an acknowledged deficit in the timely recognition and management of critical illness in paediatric patients in many African contexts.

This paper reports a mixed-methods pilot evaluation of the StART pediatric Basic Life Support course, a one-day, context-specific training with an embedded Training‑of‑Trainers model implemented in hospitals in Uganda, Nigeria, and Rwanda. Among 134 multidisciplinary participants, knowledge scores improved significantly, all passed a BLS skills assessment, and follow‑up surveys suggested increased confidence, better teamwork, and early application of skills, leading the authors to conclude that StART is feasible, acceptable, and potentially sustainable, while highlighting the need for longer-term evaluations of skill retention, institutional uptake, and patient outcomes.

Major comments

The work describes a novel educational intervention developed in response to a clearly identified need by a team including members with varied skillsets with a clear understanding and experience of the context. There are clear and important indicators of how and why this intervention differs from a number of similar interventions. As such it is both important and original.

The framework for development is briefly described. The intervention itself is briefly described and should be expanded upon in order to assist readers with understanding the intervention and its implementation as well as to understand sustainability. Please explain the following:

- What equipment is required to run the workshop, did each site already have the equipment or was it necessary to procure it and if so, how was this facilitated? Does each site have easy access to the equipment for future courses?
- What venue is used? Is it somewhere distant from the clinical setting? This is important in terms of getting participants to a venue in a timely and cost-effective manner as well as ensuring they are able to remain in the training for the day and not be called back into clinical service (as can happen when a training is delivered in the participant’s hospital setting). If it is held at the hospital setting was the bracketed point a barrier to full participation, if not, how was this addressed?
- How were the participants for the train-the-trainers identified? Had they had previous training and experience in didactics or skills delivery training? Does this reduce the amount of training required to deliver the workshop independently?

The assessment strategy is well outlined, I appreciated Table 2. This paper provides a clear outline of a pragmatic approach to assessment of impact using freely available tools with low friction uptake (including the use of Google Forms and WhatsApp links) modalities that reduce cost and environmental impact of paper-based systems and are easily used in the increasingly digitally connected African setting. The relatively simple data collection and analysis methods described are easily reproducible and accessible to centres with limited research resources such as many of the settings in which this research is conducted.

Did you consider repeating the knowledge and skills test at the 4–6-week mark to test retention? Although this may be too early to detect decay it would add to the assessment of impact. I think it would be useful to reflect on the reasons for not doing this in the discussion.

For the discussion: I have already mentioned a number of potential barriers I would like to see discussed (were they a barrier or not, how were they overcome?). An additional one to address is this: Previous work on interprofessional education and multidisciplinary team training has shown that entrenched professional hierarchies and power differentials between health professions can reduce psychological safety, inhibit speaking‑up behaviours, and limit the active participation and engagement of some professional groups during joint training activities. Was this your experience with the training? Did you use strategies to mitigate against this?

The failure of one site to conduct further trainings is disappointing but there may be some valuable lessons here. Can you expand on the reasons for failure to identify the barriers to success here?

Minor comments

Please address the following:

Line 38: Anesthesia provider: please define what is meant by this term. There are several cadres of anesthesia provider in each country mentioned. Physician providers may be specialist or medical officer, non-physician providers may be nurses, clinical officers or hold a BSc degree among other qualifications. Were the anesthesia providers in your multidisciplinary team who developed the workshop the same cadre as those for whom the workshop was developed? Were the participant physicians in your workshop anaesthesia providers, or were all anaesthesia providers non-physician? Please also elaborate on your target audience for the intervention. Were the following included: emergency room physicians and nurses, surgery, ward doctors and nurses?

Line 337: figure 2 is missing, please provide this

Thank you again for the opportunity to review this interesting and valuable paper. I would like to read the revised manuscript prior to publication please.

*Assoc Prof Rebecca Gray*

*17 March 2027*
