# Supplementary material for: Pediatric assessment and early resuscitation capacity building: Multi-country implementation of the StART Training-of-Trainers Model in Rwanda, Uganda, and Nigeria
Source: PLOS Glob Public Health. 2026 Aug 14;6(8):e0006137. doi: 10.1371/journal.pgph.0006137 (PMC13475823; doi:10.1371/journal.pgph.0006137)
Supplement: Supplementary file 11 [file pgph.0006137.s012.docx]

**Manuscript Title:**
*Pediatric assessment and early resuscitation capacity building: Implementation of the StART Training-of-Trainers Model in Rwanda, Uganda, and Nigeria*

**Response to Reviewers**

We thank the reviewer and the editorial team for their helpful and constructive comments. We have revised the manuscript in response to the points raised. The changes are shown in the marked-up manuscript and included in the clean revised version.

**Minor Comments Requiring Revision**

**1. Further clarification of assessment standardisation**

**Reviewer comment:**
The expanded description of the BLS skills assessment is helpful and addresses most prior concerns. However, given the reported 100% competency achievement, it would strengthen confidence in assessment consistency if the Methods briefly clarified how inter-rater consistency was supported across sites. For example, please specify whether assessors used explicit standardised scoring criteria or whether calibration or demonstration exercises were undertaken before assessment, and whether faculty oversight included direct moderation of scoring decisions. This would fully address potential concerns regarding assessor subjectivity.

**Response:**
Thank you for this comment. We have revised the Methods section to clarify how consistency in the practical BLS skills assessment was supported across sites. We now state that course trainers conducting assessments used the same structured checklist and predefined competency criteria. During the Training-of-Trainers phase, new trainers received demonstration, practice, feedback, and coaching in assessment delivery, including discussion of checklist items and scoring considerations. We also clarified that faculty provided oversight and mentorship during initial course delivery, including observation of assessment processes and discussion of scoring decisions where needed. In the limitations section, we added that formal inter-rater reliability testing was not undertaken.

**2. Refine framing of downstream clinical relevance**

**Reviewer comment:**
The discussion appropriately avoids overstatement regarding patient-level impact. To further strengthen the translational context, consider citing one or two LMIC studies demonstrating that improved provider recognition and resuscitation capability has been associated with improved process measures or clinical outcomes (e.g., earlier escalation, reduced deterioration, or improved survival). This would better contextualise the potential relevance of the intervention while maintaining appropriate caution regarding causal inference.

**Response:**
Thank you for this suggestion. We have revised the Discussion to add further context on the possible clinical relevance of StART, while avoiding overstatement of patient-level impact. We added two LMIC pediatric emergency care studies from Malawi and Mozambique, where structured pediatric emergency triage and treatment approaches, including ETAT-based training, were associated with reductions in pediatric inpatient or emergency department mortality. We also clarified that patient-level outcomes were not measured in this evaluation and that causal links with patient outcomes cannot be drawn from our study.

**3. Ensure consistent framing of self-reported follow-up outcomes**

**Reviewer comment:**
Several sections refer to improvements in teamwork and clinical application. As these outcomes derive from participant self-report rather than direct observation, please ensure wording is consistently framed as participant-reported application or perceived practice change throughout the abstract, discussion, and conclusions. This would further align interpretation with the study design.

**Response:**
Thank you for this comment. We have revised the Abstract, Discussion, and Conclusions so that follow-up outcomes are consistently described as participant-reported or perceived changes rather than directly observed clinical outcomes. This includes wording related to application of learning, teamwork, readiness, and behavior change. We also retained the limitation noting that follow-up outcomes were based on participant self-report rather than direct observation of clinical practice.

**4. Editorial and formatting comments**

**Reviewer/editor comment:**
Page 14: “This to assess participants satisfaction” should be revised to: “This was to assess participants’ satisfaction.” Please ensure consistent capitalisation and hyphenation of Training-of-Trainers throughout. Please verify that all figures and tables are cited sequentially and consistently in the main text. Please standardise spelling and stylistic conventions throughout according to journal house style.

**Response:**
Thank you. We have corrected the sentence to “This was to assess participants’ satisfaction.” We have also reviewed the manuscript for consistent capitalization and hyphenation of Training-of-Trainers/ToT terminology, checked figure and table citations for sequential and consistent use in the main text, and standardized spelling, hyphenation, and stylistic conventions throughout.
