# Supplementary material for: Pediatric assessment and early resuscitation capacity building: Multi-country implementation of the StART Training-of-Trainers Model in Rwanda, Uganda, and Nigeria
Source: PLOS Glob Public Health. 2026 Aug 14;6(8):e0006137. doi: 10.1371/journal.pgph.0006137 (PMC13475823; doi:10.1371/journal.pgph.0006137)
Supplement: Peer review history

# Peer review: Decision Letter 0

**DOI:** 10.1371/journal.pgph.0006137.r001

**Authors:** Barnabas Alayande

5 Apr 2026

PGPH-D-26-00651

Pediatric assessment and early resuscitation capacity building: Implementation of the StART Training-of-Trainers Model in Rwanda, Uganda, and Nigeria

PLOS Global Public Health

Dear Dr. Silvers,

Thank you for submitting your manuscript to PLOS Global Public Health. After careful consideration, we feel that it has merit but does not fully meet PLOS Global Public Health’s publication criteria as it currently stands. Therefore, we invite you to submit a revised version of the manuscript that addresses the points raised during the review process.

==============================

Thank you for this very policy -relevant submission on 'Pediatric assessment and early resuscitation capacity building: Implementation of the StART Training-of-Trainers Model in Rwanda, Uganda, and Nigeria.' All changes suggested by the reviewers are required for acceptance. Please address the discrepancy in the reported effect size, and acknowledge noted limitations. Also discuss generalizability in the light of Rwanda's sample.

Please address each reviewer comment point by point and apply suggestions.

With gratitude

==============================

Please submit your revised manuscript by May 05 2026 11:59PM. If you will need more time than this to complete your revisions, please reply to this message or contact the journal office at globalpubhealth@plos.org. When you're ready to submit your revision, log on to https://www.editorialmanager.com/pgph/ and select the 'Submissions Needing Revision' folder to locate your manuscript file.

Please include the following items when submitting your revised manuscript:

- A letter that responds to each point raised by the editor and reviewer(s). You should upload this letter as a separate file labeled 'Response to Reviewers'.
- A marked-up copy of your manuscript that highlights changes made to the original version. You should upload this as a separate file labeled 'Revised Manuscript with Track Changes'.
- An unmarked version of your revised paper without tracked changes. You should upload this as a separate file labeled 'Manuscript'.

Guidelines for resubmitting your figure files are available below the reviewer comments at the end of this letter.

As the corresponding author, your ORCID iD is verified in the submission system and will appear in the published article. PLOS supports the use of ORCID, and we encourage all coauthors to register for an ORCID iD and use it as well. Please encourage your coauthors to verify their ORCID iD within the submission system before final acceptance, as unverified ORCID iDs will not appear in the published article. *Only*  the individual author can complete the verification step; PLOS staff *cannot*  verify ORCID iDs on behalf of authors.

We look forward to receiving your revised manuscript.

Kind regards,

Barnabas Tobi Alayande

Academic Editor

PLOS Global Public Health

**Journal Requirements:**

1. Please include a complete copy of PLOS’ questionnaire on inclusivity in global research in your revised manuscript. Our policy for research in this area aims to improve transparency in the reporting of research performed outside of researchers’ own country or community. The policy applies to researchers who have travelled to a different country to conduct research, research with Indigenous populations or their lands, and research on cultural artefacts. The questionnaire can also be requested at the journal’s discretion for any other submissions, even if these conditions are not met. Please find more information on the policy and a link to download a blank copy of the questionnaire here: https://journals.plos.org/globalpublichealth/s/best-practices-in-research-reporting. Please upload a completed version of your questionnaire as Supporting Information when you resubmit your manuscript.

2. Please send a completed 'Competing Interests' statement, including any COIs declared by your co-authors. If you have no competing interests to declare, please state "The authors have declared that no competing interests exist". Otherwise please declare all competing interests beginning with the statement "I have read the journal's policy and the authors of this manuscript have the following competing interests:"

3. Please amend your detailed Financial Disclosure statement. This is published with the article. It must therefore be completed in full sentences and contain the exact wording you wish to be published.

i. Please clarify all sources of financial support for your study. List the grants, grant numbers, and organizations that funded your study, including funding received from your institution. Please note that suppliers of material support, including research materials, should be recognized in the Acknowledgements section rather than in the Financial Disclosure.

ii. State the initials, alongside each funding source, of each author to receive each grant. For example: "This work was supported by the National Institutes of Health (####### to AM; ###### to CJ) and the National Science Foundation (###### to AM)."

iii. State what role the funders took in the study. If the funders had no role in your study, please state: “The funders had no role in study design, data collection and analysis, decision to publish, or preparation of the manuscript.”

iv. If any authors received a salary from any of your funders, please state which authors and which funders.

4. We note that you have indicated that there are restrictions to data sharing for this study. For studies involving human research participant data or other sensitive data, we encourage authors to share de-identified or anonymized data. However, when data cannot be publicly shared for ethical reasons, we allow authors to make their data sets available upon request. For information on unacceptable data access restrictions, please see http://journals.plos.org/plosone/s/data-availability#loc-unacceptable-data-access-restrictions.

Before we proceed with your manuscript, please address the following prompts:

a) If there are ethical or legal restrictions on sharing a de-identified data set, please explain them in detail (e.g., data contain potentially identifying or sensitive patient information, data are owned by a third-party organization, etc.) and who has imposed them (e.g., a Research Ethics Committee or Institutional Review Board, etc.). Please also provide contact information for a data access committee, ethics committee, or other institutional body to which data requests may be sent.

b) If there are no restrictions, please upload the minimal anonymized data set necessary to replicate your study findings to a stable, public repository and provide us with the relevant URLs, DOIs, or accession numbers. Please see http://www.bmj.com/content/340/bmj.c181.long for guidelines on how to de-identify and prepare clinical data for publication. For a list of recommended repositories, please see https://journals.plos.org/plosone/s/recommended-repositories. You also have the option of uploading the data as Supporting Information files, but we would recommend depositing data directly to a data repository if possible.

Please update your Data Availability statement in the submission form accordingly.

5. Please provide separate figure files in .tif or .eps format.

For more information about figure files please see our guidelines:

https://journals.plos.org/globalpublichealth/s/figures

https://journals.plos.org/globalpublichealth/s/figures#loc-file-requirements

6. We have noticed that you have uploaded Supporting Information files, but you have not included a list of legends. Please add a full list of legends for your Supporting Information files after the references list.

If the reviewer comments include a recommendation to cite specific previously published works, please review and evaluate these publications to determine whether they are relevant and should be cited. There is no requirement to cite these works unless the editor has indicated otherwise.

Please review your reference list to ensure that it is complete and correct. If you have cited papers that have been retracted, please include the rationale for doing so in the manuscript text, or remove these references and replace them with relevant current references. Any changes to the reference list should be mentioned in the rebuttal letter that accompanies your revised manuscript. If you need to cite a retracted article, indicate the article’s retracted status in the References list and also include a citation and full reference for the retraction notice.

**Additional Editor Comments (if provided):**

[Note: HTML markup is below. Please do not edit.]

Reviewers' comments:

Reviewer's Responses to Questions

**Comments to the Author**

1. Does this manuscript meet PLOS Global Public Health’s publication criteria?

Reviewer #1: Yes

Reviewer #2: Yes

**********

2. Has the statistical analysis been performed appropriately and rigorously?

Reviewer #1: Yes

Reviewer #2: Yes

**********

3. Have the authors made all data underlying the findings in their manuscript fully available (please refer to the Data Availability Statement at the start of the manuscript PDF file)?

The PLOS Data policy

Reviewer #1: Yes

Reviewer #2: Yes

**********

4. Is the manuscript presented in an intelligible fashion and written in standard English?

Reviewer #1: Yes

Reviewer #2: Yes

**********

**Reviewer #1:**  Thank you for the opportunity to review the submission for PLOS Global Public Health:

“Pediatric assessment and early resuscitation capacity building: Implementation of the

StART Training-of-Trainers Model in Rwanda, Uganda, and Nigeria”

This valuable paper outlines the process of development, implementation and assessment of impact of a structured one-day health education intervention training for LMICs that integrates pediatric assessment, early intervention, and high-quality resuscitation skills; developed in response to an acknowledged deficit in the timely recognition and management of critical illness in paediatric patients in many African contexts.

This paper reports a mixed-methods pilot evaluation of the StART pediatric Basic Life Support course, a one-day, context-specific training with an embedded Training‑of‑Trainers model implemented in hospitals in Uganda, Nigeria, and Rwanda. Among 134 multidisciplinary participants, knowledge scores improved significantly, all passed a BLS skills assessment, and follow‑up surveys suggested increased confidence, better teamwork, and early application of skills, leading the authors to conclude that StART is feasible, acceptable, and potentially sustainable, while highlighting the need for longer-term evaluations of skill retention, institutional uptake, and patient outcomes.

Major comments

The work describes a novel educational intervention developed in response to a clearly identified need by a team including members with varied skillsets with a clear understanding and experience of the context. There are clear and important indicators of how and why this intervention differs from a number of similar interventions. As such it is both important and original.

The framework for development is briefly described. The intervention itself is briefly described and should be expanded upon in order to assist readers with understanding the intervention and its implementation as well as to understand sustainability.

Please explain the following:

• What equipment is required to run the workshop, did each site already have the equipment or was it necessary to procure it and if so, how was this facilitated? Does each site have easy access to the equipment for future courses?

• What venue is used? Is it somewhere distant from the clinical setting? This is important in terms of getting participants to a venue in a timely and cost-effective manner as well as ensuring they are able to remain in the training for the day and not be called back into clinical service (as can happen when a training is delivered in the participant’s hospital setting). If it is held at the hospital setting was the bracketed point a barrier to full participation, if not, how was this addressed?

• How were the participants for the train-the-trainers identified? Had they had previous training and experience in didactics or skills delivery training? Does this reduce the amount of training required to deliver the workshop independently?

The assessment strategy is well outlined, I appreciated Table 2. This paper provides a clear outline of a pragmatic approach to assessment of impact using freely available tools with low friction uptake (including the use of Google Forms and WhatsApp links) modalities that reduce cost and environmental impact of paper-based systems and are easily used in the increasingly digitally connected African setting. The relatively simple data collection and analysis methods described are easily reproducible and accessible to centres with limited research resources such as many of the settings in which this research is conducted.

Did you consider repeating the knowledge and skills test at the 4–6-week mark to test retention? Although this may be too early to detect decay it would add to the assessment of impact. I think it would be useful to reflect on the reasons for not doing this in the discussion.

For the discussion: I have already mentioned a number of potential barriers I would like to see discussed (were they a barrier or not, how were they overcome?). An additional one to address is this: Previous work on interprofessional education and multidisciplinary team training has shown that entrenched professional hierarchies and power differentials between health professions can reduce psychological safety, inhibit speaking‑up behaviours, and limit the active participation and engagement of some professional groups during joint training activities. Was this your experience with the training? Did you use strategies to mitigate against this?

The failure of one site to conduct further trainings is disappointing but there may be some valuable lessons here. Can you expand on the reasons for failure to identify the barriers to success here?

Minor comments

Please address the following:

- Line 38: Anesthesia provider: please define what is meant by this term. There are several cadres of anesthesia provider in each country mentioned. Physician providers may be specialist or medical officer, non-physician providers may be nurses, clinical officers or hold a BSc degree among other qualifications. Were the anesthesia providers in your multidisciplinary team who developed the workshop the same cadre as those for whom the workshop was developed? Were the participant physicians in your workshop anaesthesia providers, or were all anaesthesia providers non-physician? Please also elaborate on your target audience for the intervention. Were the following included: emergency room physicians and nurses, surgery, ward doctors and nurses?

- Line 337: figure 2 is missing, please provide this

Thank you again for the opportunity to review this interesting and valuable paper. I would like to read the revised manuscript prior to publication please.

**Reviewer #2:**  Thank you for submitting this important and policy-relevant manuscript. This study addresses a significant gap in paediatric emergency care capacity in low-resource settings through evaluation of a context-specific BLS programme with an embedded Training-of-Trainers model. The emphasis on locally led implementation and sustainability is a notable strength and aligns with current priorities in global health systems strengthening.

Major Comments (requiring revision)

1. Effect size inconsistency (critical) There is a discrepancy in the reported effect size (Cohen’s d = 1.5 in the Results vs. 2.5 in the Discussion). This should be verified and corrected to ensure consistency and accuracy throughout the manuscript.

2. Interpretation of follow-up data and generalisability The 4–6 week follow-up response rate of 67% is acceptable; however, the potential for response bias should be explicitly acknowledged, as participants with more positive experiences may have been more likely to respond.

The short follow-up period also limits conclusions regarding sustained behavioural change and skill retention, which are known to decay over time following resuscitation training. This limitation should be more clearly articulated.

The relatively small sample size in Rwanda (n = 18), and the absence of independent local training at this site, further limit conclusions regarding scalability in smaller institutions and should be discussed.

In addition, the use of convenience sampling for site selection may limit external validity and should be acknowledged as a source of potential selection bias.

3. Skills assessment outcomes (100% pass rate) The reported 100% pass rate in the BLS skills assessment requires further clarification. While this may reflect effective training, it raises the possibility of ceiling effects or limited discriminatory capacity of the assessment.

Please clarify:

• Whether assessors were standardised and/or trained

• Whether assessments were conducted independently or with blinding

• Whether any participants required remediation or repeat assessment

• How competency thresholds were defined

This will allow readers to better assess the validity and robustness of the skills evaluation.

4. Link to patient outcomes The manuscript appropriately highlights the need for future evaluation of patient outcomes. To strengthen the discussion, the authors should briefly contextualise how improvements in knowledge and early resuscitation skills may translate into clinical outcomes, with reference to existing evidence from LMIC settings where possible.

5. Subgroup analyses and statistical power Subgroup analyses by country, profession, and trainer type are informative; however, it is unclear whether these analyses were adequately powered to detect meaningful differences. This limitation should be acknowledged.

Minor Comments (for clarity and completeness)

• Abstract: Consider including the number of ToT completers (n = 14) and the follow-up response rate (67%) to improve transparency.

• Methods: The MCQ tests are described as “parallel forms.” Please clarify whether equivalence was formally assessed beyond the pilot group.

• Results: Ensure that percentages in tables are internally consistent and account for minor rounding discrepancies.

• Discussion: The COM-B framework is well integrated; consider briefly outlining how future iterations of the programme could address key barriers identified (e.g., equipment limitations).

• Editing: Correct minor typographical errors (e.g., “Study asim”) and ensure consistency in the running title and referencing of tables and figures.

Strengths

• Locally developed, context-specific training model addressing a critical gap in paediatric emergency care

• Strong integration of quantitative and qualitative methods

• Feasible, scalable, and sustainability-focused Training-of-Trainers model

Overall, this is a well-conducted and policy-relevant study, and I recommend minor revision.

**********

what does this mean?). If published, this will include your full peer review and any attached files.

**Do you want your identity to be public for this peer review?**  If you choose “no”, your identity will remain anonymous but your review may still be made public.

For information about this choice, including consent withdrawal, please see our Privacy Policy

Reviewer #1: **Yes:**  Associate Professor Rebecca Gray

Reviewer #2: **Yes:**  Shameer Mohamed Naleer

**********

[NOTE: If reviewer comments were submitted as an attachment file, they will be attached to this email and accessible via the submission site. Please log into your account, locate the manuscript record, and check for the action link "View Attachments". If this link does not appear, there are no attachment files.]

**Figure Resubmissions:**

Attachment Submitted filename: PLOS review.docx

# Author comment: Author response to Decision Letter 1

**DOI:** 10.1371/journal.pgph.0006137.r002

28 Apr 2026

Attachment Submitted filename: Response to Reviewers.docx

# Peer review: Decision Letter 1

**DOI:** 10.1371/journal.pgph.0006137.r003

**Authors:** Barnabas Alayande

17 May 2026

PGPH-D-26-00651R1

Pediatric assessment and early resuscitation capacity building: Implementation of the StART Training-of-Trainers Model in Rwanda, Uganda, and Nigeria

PLOS Global Public Health

Dear Dr. Silvers,

Thank you for submitting your manuscript to PLOS Global Public Health. After careful consideration, we feel that it has merit but does not fully meet PLOS Global Public Health’s publication criteria as it currently stands. Therefore, we invite you to submit a revised version of the manuscript that addresses the points raised during the review process.

==============================

Thank you for an improved version of the manuscript as noted by reviewers.

Please address all the following reviewer comments to proceed-

"Minor Comments Requiring Revision

1. Further clarification of assessment standardisation

The expanded description of the BLS skills assessment is helpful and addresses most prior concerns. However, given the reported 100% competency achievement, it would strengthen confidence in assessment consistency if the Methods briefly clarified how inter-rater consistency was supported across sites. For example, please specify whether assessors used explicit standardised scoring criteria or whether calibration or demonstration exercises were undertaken before assessment, and whether faculty oversight included direct moderation of scoring decisions.  This would fully address potential concerns regarding assessor subjectivity.

2. Refine framing of downstream clinical relevance

The discussion appropriately avoids overstatement regarding patient-level impact. To further strengthen the translational context, consider citing one or two LMIC studies demonstrating that improved provider recognition and resuscitation capability has been associated with improved process measures or clinical outcomes (e.g., earlier escalation, reduced deterioration, or improved survival). This would better contextualise the potential relevance of the intervention while maintaining appropriate caution regarding causal inference.

3. Ensure consistent framing of self-reported follow-up outcomes

Several sections refer to improvements in teamwork and clinical application. As these outcomes derive from participant self-report rather than direct observation, please ensure wording is consistently framed as participant-reported application or perceived practice change throughout the abstract, discussion, and conclusions.  This would further align interpretation with the study design.

Also address noted Minor Editorial Corrections:

Page 14: “This to assess participants satisfaction” should be revised to: “This was to assess participants’ satisfaction.” Please ensure consistent capitalisation and hyphenation of Training-of-Trainers throughout.

Please verify that all figures and tables are cited sequentially and consistently in the main text. Please standardise spelling and stylistic conventions throughout according to journal house style."

With gratitude

==============================

Please submit your revised manuscript by Jun 16 2026 11:59PM. If you will need more time than this to complete your revisions, please reply to this message or contact the journal office at globalpubhealth@plos.org. When you're ready to submit your revision, log on to https://www.editorialmanager.com/pgph/ and select the 'Submissions Needing Revision' folder to locate your manuscript file.

Please include the following items when submitting your revised manuscript:

- A letter that responds to each point raised by the editor and reviewer(s). You should upload this letter as a separate file labeled 'Response to Reviewers'.
- A marked-up copy of your manuscript that highlights changes made to the original version. You should upload this as a separate file labeled 'Revised Manuscript with Track Changes'.
- An unmarked version of your revised paper without tracked changes. You should upload this as a separate file labeled 'Manuscript'.

Guidelines for resubmitting your figure files are available below the reviewer comments at the end of this letter.

As the corresponding author, your ORCID iD is verified in the submission system and will appear in the published article. PLOS supports the use of ORCID, and we encourage all coauthors to register for an ORCID iD and use it as well. Please encourage your coauthors to verify their ORCID iD within the submission system before final acceptance, as unverified ORCID iDs will not appear in the published article. *Only*  the individual author can complete the verification step; PLOS staff *cannot*  verify ORCID iDs on behalf of authors.

We look forward to receiving your revised manuscript.

Kind regards,

Barnabas Tobi Alayande

Academic Editor

PLOS Global Public Health

**Journal Requirements:**

If the reviewer comments include a recommendation to cite specific previously published works, please review and evaluate these publications to determine whether they are relevant and should be cited. There is no requirement to cite these works unless the editor has indicated otherwise.

Please review your reference list to ensure that it is complete and correct. If you have cited papers that have been retracted, please include the rationale for doing so in the manuscript text, or remove these references and replace them with relevant current references. Any changes to the reference list should be mentioned in the rebuttal letter that accompanies your revised manuscript. If you need to cite a retracted article, indicate the article’s retracted status in the References list and also include a citation and full reference for the retraction notice.

**Additional Editor Comments (if provided):**

[Note: HTML markup is below. Please do not edit.]

Reviewers' comments:

Reviewer's Responses to Questions

**Comments to the Author**

Reviewer #1: All comments have been addressed

Reviewer #2: All comments have been addressed

**********

publication criteria?

Reviewer #1: Yes

Reviewer #2: Yes

**********

3. Has the statistical analysis been performed appropriately and rigorously?

Reviewer #1: Yes

Reviewer #2: Yes

**********

4. Have the authors made all data underlying the findings in their manuscript fully available (please refer to the Data Availability Statement at the start of the manuscript PDF file)?

The PLOS Data policy

Reviewer #1: Yes

Reviewer #2: Yes

**********

5. Is the manuscript presented in an intelligible fashion and written in standard English?

Reviewer #1: Yes

Reviewer #2: Yes

**********

**Reviewer #1:**  Thank you for your revised manuscript, it is a great addition to our literature and I look forward to seeing it published and sharing it with interested colleagues.

**Reviewer #2:**  This revised manuscript presents a well-conducted mixed-methods pilot evaluation of a contextually adapted paediatric Basic Life Support (BLS) Training-of-Trainers (ToT) intervention implemented across three sub-Saharan African hospital settings.

The study addresses an important gap in contextually relevant paediatric emergency capacity-building in low-resource settings and aligns strongly with the journal’s emphasis on equity-oriented, implementation-focused global public health research. The authors have responded constructively to the concerns raised during the initial review and have substantially strengthened the manuscript through improved methodological transparency, internally consistent effect size reporting, expanded discussion of study limitations, and additional detail regarding assessment procedures and implementation processes.

The intervention is locally designed, operationally feasible, and evaluated using an appropriately integrated mixed-methods approach. The findings provide useful preliminary evidence regarding feasibility, acceptability, knowledge acquisition, and early participant-reported application of learning in practice.

The remaining issues are minor clarifications and editorial refinements that can be readily addressed prior to publication.

Notably, the revision has improved:

1. Consistency of effect size reporting

2. Transparency regarding practical skills assessment procedures

3. Acknowledgement of methodological limitations, including convenience sampling, response bias, limited follow-up duration, and the smaller Rwanda implementation cohort

4. Clarification of implementation procedures and trainer preparation.

These revisions improve both methodological clarity and interpretive rigour.

The manuscript is now close to publication standard. I recommend acceptance following minor revision to address the points below.

Minor Comments Requiring Revision

1. Further clarification of assessment standardisation

The expanded description of the BLS skills assessment is helpful and addresses most prior concerns. However, given the reported 100% competency achievement, it would strengthen confidence in assessment consistency if the Methods briefly clarified how inter-rater consistency was supported across sites. For example, please specify whether assessors used explicit standardised scoring criteria or whether calibration or demonstration exercises were undertaken before assessment, and whether faculty oversight included direct moderation of scoring decisions.

This would fully address potential concerns regarding assessor subjectivity.

2. Refine framing of downstream clinical relevance

The discussion appropriately avoids overstatement regarding patient-level impact. To further strengthen the translational context, consider citing one or two LMIC studies demonstrating that improved provider recognition and resuscitation capability has been associated with improved process measures or clinical outcomes (e.g., earlier escalation, reduced deterioration, or improved survival). This would better contextualise the potential relevance of the intervention while maintaining appropriate caution regarding causal inference.

3. Ensure consistent framing of self-reported follow-up outcomes

Several sections refer to improvements in teamwork and clinical application. As these outcomes derive from participant self-report rather than direct observation, please ensure wording is consistently framed as participant-reported application or perceived practice change throughout the abstract, discussion, and conclusions.

This would further align interpretation with the study design.

Minor Editorial Corrections:

Page 14:

“This to assess participants satisfaction” should be revised to: “This was to assess participants’ satisfaction.”

Please ensure consistent capitalisation and hyphenation of Training-of-Trainers throughout.

Please verify that all figures and tables are cited sequentially and consistently in the main text.

Please standardise spelling and stylistic conventions throughout according to journal house style.

This work makes a valuable contribution to the literature on contextually adapted emergency care capacity-building in low-resource settings. Subject to the minor clarifications above, I support publication of this manuscript.

**********

what does this mean?). If published, this will include your full peer review and any attached files.

**Do you want your identity to be public for this peer review?**  If you choose “no”, your identity will remain anonymous but your review may still be made public.

For information about this choice, including consent withdrawal, please see our Privacy Policy

Reviewer #1: No

Reviewer #2: **Yes:**  Shameer Mohamed Naleer

**********

[NOTE: If reviewer comments were submitted as an attachment file, they will be attached to this email and accessible via the submission site. Please log into your account, locate the manuscript record, and check for the action link "View Attachments". If this link does not appear, there are no attachment files.]

**Figure Resubmissions:**

# Author comment: Author response to Decision Letter 2

**DOI:** 10.1371/journal.pgph.0006137.r004

27 May 2026

Attachment Submitted filename: Response to Reviewer Revisions.docx

# Peer review: Decision Letter 2

**DOI:** 10.1371/journal.pgph.0006137.r005

**Authors:** Julia Robinson

9 Jun 2026

Pediatric assessment and early resuscitation capacity building: Implementation of the StART Training-of-Trainers Model in Rwanda, Uganda, and Nigeria

PGPH-D-26-00651R2

Dear Dr Silvers,

We are pleased to inform you that your manuscript 'Pediatric assessment and early resuscitation capacity building: Implementation of the StART Training-of-Trainers Model in Rwanda, Uganda, and Nigeria' has been provisionally accepted for publication in PLOS Global Public Health.

Before your manuscript can be formally accepted you will need to complete some formatting changes, which you will receive in a follow up email. A member of our team will be in touch with a set of requests.

Please note that your manuscript will not be scheduled for publication until you have made the required changes, so a swift response is appreciated.

IMPORTANT: The editorial review process is now complete. PLOS will only permit corrections to spelling, formatting or significant scientific errors from this point onwards. Requests for major changes, or any which affect the scientific understanding of your work, will cause delays to the publication date of your manuscript.

If your institution or institutions have a press office, please notify them about your upcoming paper to help maximize its impact. If they'll be preparing press materials, please inform our press team as soon as possible -- no later than 48 hours after receiving the formal acceptance. Your manuscript will remain under strict press embargo until 2 pm Eastern Time on the date of publication. For more information, please contact globalpubhealth@plos.org.

Thank you again for supporting Open Access publishing; we are looking forward to publishing your work in PLOS Global Public Health.

Best regards,

Julia Robinson

Executive Editor

PLOS Global Public Health

***********************************************************

Reviewer Comments (if any, and for reference):

Reviewer's Responses to Questions

**Comments to the Author**

Reviewer #1: All comments have been addressed

Reviewer #2: All comments have been addressed

**********

publication criteria?

Reviewer #1: Yes

Reviewer #2: Yes

**********

3. Has the statistical analysis been performed appropriately and rigorously?

Reviewer #1: Yes

Reviewer #2: Yes

**********

4. Have the authors made all data underlying the findings in their manuscript fully available (please refer to the Data Availability Statement at the start of the manuscript PDF file)?

The PLOS Data policy

Reviewer #1: Yes

Reviewer #2: Yes

**********

5. Is the manuscript presented in an intelligible fashion and written in standard English?

Reviewer #1: Yes

Reviewer #2: Yes

**********

Reviewer #1: (No Response)

Reviewer #2: Thank you very much for the opportunity to review this manuscript again. This revised revision represents a significant change and a thoughtful revision - compared to the previous version. The authors have responded comprehensively to reviewer feedback and have strengthened the manuscript considerably.

In particular, the revisions have improved:

- Transparency regarding skills assessment procedures and standardisation;

- Appropriate framing of follow-up findings as participant-reported outcomes;

- Discussion of methodological limitations; and

- Overall clarity and rigour of the work.

Importantly, this manuscript provides an important and timely contribution to the literature on paediatric emergency care capacity building in low-resource settings. In particular, the Training-of-Trainers model is a particular strength, supporting sustainability and local ownership while demonstrating feasibility across diverse implementation contexts.

I have no further substantive concerns and believe the manuscript is suitable for publication.

Strengths

- Locally co-designed and contextually relevant intervention.

- Strong mixed-methods approach with meaningful integration of quantitative and qualitative findings.

- Clear focus on sustainability through the embedded Training-of-Trainers model.

- Transparent discussion of limitations and appropriate interpretation of findings.

- Important contribution to paediatric emergency care and workforce development in LMIC settings.

I recommend acceptance.

**********

what does this mean?). If published, this will include your full peer review and any attached files.

**Do you want your identity to be public for this peer review?**  If you choose “no”, your identity will remain anonymous but your review may still be made public.

For information about this choice, including consent withdrawal, please see our Privacy Policy

Reviewer #1: **Yes:**  Rebecca M Gray

Reviewer #2: **Yes:**  Shameer M Naleer

**********
